# FOXG1 Hierarchically Shapes Synaptic Functions in Striatal iSPNs and Contributes to ASD Etiology

**DOI:** 10.1007/s12264-025-01573-3

**Published:** 2026-02-02

**Authors:** Baoshen Zhang, Daxiang Xu, Shuangshuang Dong, Pei Zhu, Pengfei Jiang, Jie Sun, Junhua Liu, Huanxin Chen, Chunjie Zhao

**Affiliations:** 1 Key Laboratory of Developmental Genes and Human Diseases, Ministry of Education, School of Medicine, Southeast University, Nanjing, 210009 China; 2 Department of Anesthesiology, Surgery and Pain Management & Key Laboratory of Clinical Science and Research, Zhongda Hospital, Nanjing, 210009 China; 3 Huzhou Third Municipal Hospital, The Affiliated Hospital of Huzhou University, Huzhou, 313000 China

**Keywords:** Autism spectrum disorder, Striatum, Indirect pathway spiny projection neurons, AMPAR, Synaptogenesis, Synaptic transmission, Dendritic development, Forkhead box G1

## Abstract

Autism spectrum disorder (ASD) pathophysiology often involves striatal dysfunction, yet the underlying mechanisms remain unclear. Mutations in Forkhead box G1 (*FOXG1*) cause FOXG1 syndrome, a condition sharing core ASD features. Here, loss of *Foxg1* in the indirect pathway spiny projection neurons (iSPNs) in mice recapitulates ASD symptoms, including social, language, and fine movement deficits. *Foxg1* deficiency causes dendritic simplification, spine reduction, and impairs excitatory synaptic transmission. Transcriptome reveals that FOXG1 drives gene networks to multidimensionally control synaptic functions from spine morphogenesis, synaptic maturation, ion transmembrane transport, glutamate receptor clustering, to neurotransmitter release and synaptic transmission. Importantly, FOXG1 directly activates the transcription of α-amino-3-hydroxy-5-methyl-4-isoxazolepropionic acid receptor (AMPAR) subunits, and pharmacological potentiation of AMPAR activity normalizes synaptic function and rescues behavioral deficits. Our study provides a new perspective on the relationship between FOXG1 and ASD etiology in iSPNs and suggests the potential of AMPAR activation as a therapeutic intervention for ASD and FOXG1 Syndrome.

**Supplementary Information:**

The online version contains supplementary material available at 10.1007/s12264-025-01573-3.

## Introduction

The striatum is the gateway to the basal ganglia, an ensemble of subcortical structures where information for social behavior, linguistic processing, motor coordination, and affective regulation converge [1–5]. Striatal dysfunction has been strongly implicated in a variety of psychiatric disorders defined by inflexible and maladaptive behaviors such as Autism Spectrum Disorder (ASD) [6, 7]. Structural and functional magnetic resonance imaging (MRI) studies across multiple ASD cohorts consistently demonstrate striatal hypertrophy and altered frontal cortical-striatal connectivity [8–11], pointing to concurrent structural and circuit-level alterations in the striatum. Beyond corticostriatal systems disturbances in ASD [12–16], the amygdalostriatal pathway has risen to prominence as a modulator of social avoidance behaviors relevant to ASD, as evidenced by optogenetic activation of basolateral amygdala-nucleus accumbens [17], a component of the ventral striatum. In spite of the establishment of the dysfunctional striatal networks as a central hub in ASD pathogenesis, molecular underpinnings remain elusive.

The striatum consists of two main circuits, the so-called direct and indirect pathways [18]. Classical models postulate that these two pathways exert opposing effects, yet their cooperative and balanced activity is essential for proper neural function [19–21]. Indirect pathway spiny projection neurons (iSPNs) expressing dopamine D2 receptors, integrate glutamatergic inputs and then project predominantly to the globus pallidus externa to modulate motor control, reward processing, and cognitive functions [22, 23]. Accumulating evidence shows that iSPNs are involved in behavioral abnormalities associated with ASD. It has been reported that animal models with attenuated iSPNs synaptic activity exhibit concomitant social interaction deficits and stereotypic behaviors [24–26]. On the other hand, iSPN hyperactivation is demonstrated to drive core ASD-like repetitive behaviors as well [27]. Despite the bidirectional dysregulation of iSPN activity in ASD pathologies, the molecular mechanisms underlying iSPN synaptic transmission dysfunction remain to be elucidated.

Forkhead box G1 (FOXG1) functions as a conserved forkhead box transcription factor essential for forebrain development [28–35]. Mutations in *FOXG1* cause FOXG1 syndrome, a condition sharing core ASD features, manifesting social deficits and language impairments [36–38]. Notably, *Foxg1*-null mutants exhibit an absence of ventral forebrain structures, including the striatum, highlighting its potential role in orchestrating ventral telencephalic development [30]. In this study, we find that loss of *Foxg1* in iSPNs leads to ASD-like behaviors alongside dendritic developmental abnormalities and a pronounced reduction in excitatory synaptic transmission. We uncover that FOXG1 directly activates *Gria1*, *Gria2*, and *Gria3,* which encode core AMPAR subunits. Importantly, pharmacological enhancement of AMPAR activity rescues synaptic deficits and ameliorates behavioral abnormalities in *Foxg1* conditional knockout (cKO) mice. Beyond revealing the role of FOXG1 in striatal iSPNs, our findings provide novel insights into the pathological mechanisms and treatments underlying FOXG1 syndrome as well as ASD.

## Materials and Methods

### Animals

All experiments were carried out using C57BL/6J background mice. We used *Foxg1*^fl/fl^ mice and lines for fluorescence reporter (*Ai9*, stock number: 007905, Jackson Laboratory, USA) and for Cre (*Drd2-Cre*, stock number: 017263, MMRRC, Davis, USA). In this article, when mentioning *Foxg1* cKO mice, *Drd2-Cre;Foxg1*^fl/fl^*;Ai9* is implied as the default, with *Foxg1*^fl/fl^ being the default for the controls. Behavioral tests were conducted exclusively in male mice, while other experiments utilized mixed-sex cohorts. To ensure the circadian rhythm of laboratory mice, we housed the mice in a controlled environment with 12 h of darkness and 12 h of light. The relevant experiments in this study were conducted only after obtaining approval from the Animal Ethics Committee of Southeast University (approval No. #20211014001), during which we strictly adhered to experimental protocols and requirements to respect each laboratory mouse.

### Immunofluorescence and *In Situ* Hybridization

Following transcardial perfusion [20 mL phosphate-buffered saline (PBS) + 40 mL 4% paraformaldehyde (PFA)], brain tissues from 2-month-old *Foxg1* cKO and control mice were post-fixed, sucrose-protected, and sectioned into 30 μm slices using a Leica CM3050S cryostat (Wetzlar, Germany). For immunofluorescence: (1) brain slices were permeabilized (0.2% Triton X-100/PBS for 35 min); (2) blocked with 10% serum [2 h, room temperature (RT)]; (3) incubated with anti-FOXG1 (Abcam, AB18259, 1:1000, Cambridge, UK) for 16–18 h at 4°C; (4) secondary antibody incubation (2 h, RT); (5) imaged using Olympus FV1000 confocal microscope (Tokyo, Japan). For *in situ* hybridization: (1) digoxigenin (DIG)-labeled probes were polymerase chain reaction (PCR)-amplified (Table S1 primers); (2) section hybridization (65°C overnight); (3) anti-DIG-alkaline phosphatase (AP) incubation (2 h, RT); (4) colorimetric development.

### Western Blot

We extracted proteins from the striatal region and separated them using self-prepared gels [10% sodium dodecyl sulfate (SDS)-polyacrylamide gel electrophoresis]. After successful protein separation, we carefully transferred them onto the membrane. The blocking step was carried out for 2 h using 5% nonfat dry milk prepared in tris-buffered saline with Tween 20 (TBST) buffer (20 mmol/L Tris-HCl, pH 7.5, 150 mmol/L NaCl, and 0.1% Tween 20). For immunodetection, membranes were first incubated with primary antibodies (anti-FOXG1, Abcam, AB18259, 1:1000, Cambridge, UK; anti-GluR1, AiFang biological, AFW1826, 1:1000, Nanjing, China; anti-GluR2, AiFang biological, AF301488, 1:2000, Nanjing, China; anti-GluR3, CST, D47E3, 1:1000, Danvers, USA; anti-GAPDH, CST, D16H11, 1:5000) at 4°C overnight, followed by 2-h incubation at 37°C with horseradish peroxidase (HRP)-conjugated anti-rabbit secondary antibody.

### Behavioral Experiments

#### Open Field Test

The experiments were conducted in a 40 cm × 40 cm box, and the activity of each mouse was recorded for 30 min using high-resolution digital cameras. Spontaneous locomotion was assessed by measuring average velocity and total distance traveled, analyzed using EthoVision software from Noldus company (Wageningen, Netherlands).

#### Three Chamber Social Behavior Test

Social ability and response to social novelty were evaluated using a three-chamber apparatus. Before behavioral testing, all 2-month-old subjects were required to acclimate to the testing environment for at least 2 weeks. Age-matched target mice (“Stranger 1” and “Stranger 2”) were acclimated for 2 days. Test comprised three phases: (1) habituation, where mice freely explored the apparatus for 5 min; (2) social ability, 10-min free exploration between left (empty cage) and right (stranger 1 cage); (3) social novelty, the experimental mouse was carefully introduced into the central compartment, while a new unfamiliar conspecific (stranger 2) replaced the vacant enclosure. Subsequently, the test subject had free access to investigate all three chambers for an additional 10 min. We utilized the EthoVision system for real-time experimental recording and precise data analysis.

#### Ultrasonic Vocalization (USV) Test

Ultrasonic vocalization (USV) from postnatal day 4 (P4) and postnatal day 7 (P7) mouse pups, selected to model early-life and pre-adolescent stages in humans, was recorded using an Avisoft UltraSoundGate 116Hm microphone (Avisoft Bioacoustics, Berlin, Germany) and analyzed with Avisoft Bioacoustic software (Avisoft Bioacoustics, Berlin, Germany). To minimize maternal interference and ensure individual assessment, each pup was separated from the dam 10 min prior to testing and individually placed in a sound-attenuating anechoic chamber maintained at a constant temperature (22–24°C). Vocalizations within the frequency range of 20–250 kHz were recorded for 5 min per pup, with the microphone placed 5 cm above the test pups to ensure optimal signal capture. Post-recording, pups were toe-tattooed for subsequent genotype identification and promptly returned to the dam to minimize stress. The number and duration of USVs were quantified using Avisoft Bioacoustic software.

#### Balance Beam Test

The skilled movement of the *Foxg1* cKO and control mice was evaluated using a balance beam apparatus. The assessment setup comprised a 100-cm-long, 1-cm-wide cuboid beam elevated 100 cm above the ground. One end of the beam was attached to a sealed, dark plastic box with an entrance, serving as the final destination. Mice were initially positioned at the starting point of the beam, facing the target box, but positioned away from it. The time taken for each mouse to traverse an 80 cm distance along the beam and reach the target box was manually recorded by two different experimenters. To ensure consistency, each mouse was given three trials, with a 10-min rest interval between trials. The average traversal time was calculated and used for statistical analysis.

#### Sunflower Seeds Opening Test

The sunflower seeds opening test was conducted to evaluate fine motor skills and dexterity in mice [39]. Researchers introduced an individual mouse into a testing chamber where 30 sunflower seeds were clustered on a single corner. To assess seed-opening behavior, mice were required to grasp the seeds with their forepaws, peel the shells, and extract the kernels. The primary outcome measure was the cumulative count of fully decorticated seeds at 3, 8, 12, and 24 h. To ensure consistency, the arena was cleaned between trials to remove residual shells and prevent olfactory cues.

#### Nest Building Test

Nest building performance was investigated using a previously established protocol [40]. Two-month-old mice were individually housed in sterile cages for 16–18 h, each provided with a 3 g untreated cotton nesting material. After 16–18 h, the amount of unused nestlet was weighed, and the utilization rate was calculated as the ratio of used nestlet mass to the initial nestlet mass (3 g). High-resolution images of the nests were also captured to evaluate nest quality.

#### Accelerating Rotarod Test

Motor learning function evaluation was performed using a progressively accelerating rotarod system. Two-month-old mice were habituated to the experimental environment for half an hour before testing. Subjects were sequentially positioned in individual compartments of a 5-station rotarod device (ITCC Life Science, Model Series 8, Sanford, USA), where rotation speed gradually increased from 4 to 40 revolutions every minute during a 5-min period. Fall latency was automatically documented. The test was repeated over three consecutive days, with four daily repetitions spaced by 20-min rest periods to prevent exhaustion. Between trials, the rotarod lanes were cleaned to remove any residual scent or debris that could influence subsequent tests.

### Quantitative Real-Time PCR (qPCR)

Total RNA extraction from the P30 murine striatal region was performed with the SPARKeasy RNA Kit (SparkJade, AC0202, Shanghai, China). We examined RNA quality *via* NanoDrop spectrophotometry (Thermo Fisher Scientific, Waltham, USA), maintaining A260/A280 ratios of 1.8–2.0. Following genomic DNA elimination, reverse transcription was conducted on 1 μg RNA employing random hexamers and oligo (dT) primers as per standardized protocols. SYBR^®^ Green-based qPCR analysis was executed on a Step One-Plus platform (Applied Biosystems, Foster, USA) using Roche master mix (04707516001). Reaction volumes (20 μL) comprised: 10 μL SYBR Green Mix, 0.5 μmol/L up/down primer, and 1 μL complementary DNA (cDNA). The PCR protocol consisted of an initial denaturation step at 95°C for 10 min, followed by 40 cycles of denaturation (95°C, 15 s) and annealing/extension (60°C, 60 s). Amplification specificity was validated through melt curve analysis. Gene expression quantification employed the 2^−ΔΔCt^ approach, normalized to GAPDH (primer sequences shown in Table S2).

### Sparse Labeling and Morphological Analyses

To analyze neuronal morphology in 2-month-old *Foxg1* cKO and control mice, we employed sparse labeling combined with detailed morphological assessment. Cre-inducible adeno-associated viral vectors (Braincase, AAV-CSSP-YFP-2E4) encoding green fluorescent protein were microinjected into the dorsolateral striatum (stereotaxic coordinates: anterior-posterior +0.72 mm, medial-lateral ±2.32 mm, dorsal-ventral −3.00 mm from Bregma). Viral delivery consisted of 100 nL total volume administered at 20 nL/min using a precision infusion system. Following a 21-day expression period, transcardial perfusion was performed using PBS and 4% PFA. Brains underwent 12–16 h post-fixation, followed by cryoprotection in 30% sucrose/PBS solution. Coronal sections containing the striatum (60 μm thickness) were cut using a cryostat and imaged on FV1000 with a 60× objective. *Z*-stacks were acquired at 1 μm intervals for subsequent analysis.

For Sholl analysis, 2D maximum intensity projections of individual iSPN were generated, and dendritic arborization was quantified using the ImageJ Sholl Analysis plugin. Concentric rings were spaced at 10 μm intervals from the soma, and the number of dendritic crossings with each concentric circle was documented. Dendritic spine morphology was assessed by counting spines along a 10 μm segment of secondary dendrites.

### Whole-Cell Patch-Clamp Recordings

Animals of either sex used in electrophysiological recording were 28–42 days old. Brain slices were collected from deeply anesthetized mice following transcardial perfusion. Using a Leica VT1200S vibratome (Wetzlar, Germany), 300-μm coronal slices were obtained in oxygenated, ice-cold protective solution containing (in mmol/L): 25 NaHCO_3_, 3.5 KCl, 80 NaCl, 0.5 CaCl_2_, 1.25 NaH_2_PO_4_, 4.5 MgSO_4_, 90 sucrose, and 10 glucose. For electrophysiological recordings, slices were maintained in carbogen-saturated artificial cerebral spinal fluid (ACSF, in mmol/L: 2.5 KCl, 126 NaCl, 1.2 NaH_2_PO_4_, 10 glucose, 1.2 MgSO_4_, 26 NaHCO_3_, and 2.4 CaCl_2_, pH 7.3–7.4, 300–310 mOsm) with sequential incubation: 34°C for 30 min followed by 1-h equilibration at RT. Whole-cell patch-clamp recordings were acquired using borosilicate glass electrodes (Sutter BF-150-86-10, CA, USA, 5–8 MΩ) filled with intracellular solution (in mmol/L): 115 K-gluconate, 20 KCl, 1.5 MgCl_2_, 10 HEPES, 10 EGTA, 2 ATP, 0.5 GTP, and 10 phosphocreatine (pH 7.25, 280–290 mOsm).

Recording was made from AI9-positive iSPNs of the striatum and was completed within 6 h of slice preparation to ensure optimal tissue viability. For miniature excitatory postsynaptic currents (mEPSCs) recordings, neurons were voltage-clamped at −75 mV in the presence of synaptic blockers including 1 μmol/L tetrodotoxin (TTX, Chengdu Navigator, A0224, Chengdu, China), 20 μmol/L gamma-aminobutyric acid type-A receptor (GABA-A receptor) antagonist bicuculline (MCE, HY.100783, Monmouth Junction, NJ, USA), and 50 μmol/L N-methyl-D-aspartate (NMDA) receptor blocker D-AP5 (MCE, HY-100714A). To assess the AMPAR/NMDAR ratio, both AMPAR- and NMDAR-mediated evoked excitatory postsynaptic currents (eEPSCs) were evoked using the same stimulation protocol and intensity. Using a holding potential of −75 mV in this article, we recorded pharmacologically isolated AMPAR-mediated EPSCs to measure rectification properties. In order to record NMDAR-mediated EPSCs, we maintained iSPNs at +40 mV to eliminate Mg^2+^ blockade. These experiments were performed in the presence of 10 µmol/L AMPAR blocker CNQX (MCE, HY-15068), using identical stimulation parameters as those employed for AMPAR-mediated excitatory postsynaptic current (AMPAR-EPSC) recordings. For quantitative comparison, we calculated the AMPAR/NMDAR ratio by comparing the average peak amplitude of AMPAR-mediated EPSCs to NMDAR-mediated EPSCs. Besides, we measured the paired pulse ratio (PPR) by delivering two consecutive stimuli separated by a 50-ms interval. This parameter was derived from the second EPSC amplitude divided by the first EPSC amplitude, averaging results from 10 to 40 trials for each recorded iSPN.

Hyperpolarizing and depolarizing current steps (ranging from −100 to +250 pA) were employed to evaluate the inherent electrophysiological characteristics of identified iSPNs, including input resistance, resting membrane potential, and action potential firing frequency. These properties were measured immediately upon break-in. Chemical reagents for patch-clamp recordings were exclusively obtained from Sigma-Aldrich, with exceptions clearly documented in the methods. The Digidata 1550B acquisition system (Molecular Devices, San Jose, USA) was configured for 2 kHz low-pass filtering with 10 kHz analog-to-digital conversion. All patch-clamp experiments were conducted with: (1) MultiClamp 700B system (Axon Instruments, Molecular Devices, San Jose, USA) for signal amplification; (2) Clampfit software (v10.6, Axon Instruments) for data analysis.

### RNA Sequencing

Total RNA was extracted from the striatal region of *Foxg1* cKO and control littermates using the SPARKeasy RNA Kit (SparkJade, AC0202). We verified RNA quality through both gel electrophoresis and quantitative analysis using a Qubit fluorometer (Waltham, MA, USA). For library preparation, we employed the Illumina TruSeq Stranded mRNA Library Prep Kit (San Diego, USA). Paired-end sequencing (2 × 150 bp) was performed on an Illumina NovaSeq 6000 platform through a service contract with Genergy Biotechnology (Shanghai, China). The bioinformatics workflow included: (1) raw read processing with Skewer for adapter trimming; (2) quality control assessment using FastQC (v0.11.2); (3) genome alignment to Genome Reference Consortium Mouse Build 38 (GRCm38) (release-98) with the Spliced Transcripts Alignment to a Reference (STAR) aligner; (4) transcript assembly and quantification *via* StringTie (v1.3.1c). Transcript abundance was quantified in FPKM (fragments per kilobase per million mapped reads) *via* Perl scripts. The differentially expressed gene analysis for RNA-seq data (DEGseq) package [employing the MA-plot-based random sampling method with random sampling (MARS) model] was used to identify differentially expressed genes (DEGs) between *Foxg1* cKO and control groups (absolute fold change >1.13, *P-*value <0.05). Functional enrichment analysis was performed in Database for Annotation, Visualization, and Integrated Discovery (DAVID), with Gene Ontology (GO) terms considered significant at *P-*value <0.05.

### Cleavage Under Targets and Tagmentation (CUT&Tag) and Identification of FOXG1-binding Site

The CUT&Tag procedure was carried out using the Hyperactive Universal CUT&Tag assay kit for Illumina (Vazyme Biotech Co., Ltd, TD903, Nanjing, China). In short, P30 nuclear suspensions from striatal neurons were bound to Concanavalin A-coated beads. The nuclei were then resuspended in antibody buffer and sequentially incubated with a primary antibody against FOXG1 (Abcam, AB18259, 1 μg) and secondary antibodies. For the control striatal neurons, the nuclear suspension was split into three samples: two replicates (replicate 1 and replicate 2) treated with FOXG1 antibody, and a negative control without antibody. Additionally, nuclei from *Foxg1* cKO striatal neurons were processed with FOXG1 antibody. Following antibody incubation, samples were exposed to pA-Tn5 transposase for tagmentation. DNA was then extracted, amplified, and purified for library preparation. Adapter trimming and quality filtering of raw FASTQ files were performed using Cutadapt (v5.2). The resulting high-quality sequencing reads were mapped to the mm10 reference genome *via* Bowtie2 (v2.5.4). Significant binding sites were identified using Model-based Analysis of ChIP-Seq 2 (MACS2) with a dynamic Poisson distribution model (*P-*value <0.01). For visualization, bigWig files were generated using deepTools’ (v3.5.5) bamCoverage, and genomic tracks were inspected with the Integrative Genomics Viewer (IGV, v2.14.1).

CUT&Tag analysis revealed FOXG1 binding sites at the promoter region of *Gria1*, *Gria2*, and *Gria3* loci. Bioinformatics assessment *via* the University of California, Santa Cruz (UCSC) Genome Browser demonstrated evolutionary conservation of these sites, with the strongest peaks localized within each gene’s promoter region. To functionally validate these findings, DNA fragments encompassing the FOXG1 binding regions (*Gria1*: 1,456 bp; *Gria2*: 2,229 bp; *Gria3*: 2,054 bp) were cloned into luciferase reporter plasmids. Each construct was co-transfected with a *Foxg1* expression plasmid into Neuro2a cells for subsequent analysis.

### Protein and Protein Interaction (PPI) Analyses

Protein and protein interaction (PPI) network analyses were performed using 89 synaptic function-associated DEGs identified through GO term analysis. Gene interactions were analyzed using STRING (v12.0, Zurich, Switzerland). We applied a stringent interaction threshold (combined score >0.700) for significance determination. The resulting network was visualized using Cytoscape (v3.9.1), with key subnetworks identified through molecular complex detection technology (MCODE) analysis. Hub genes were determined using CytoHubba’s maximal clique centrality (MCC) algorithm in Cytoscape, selecting the top 10 candidates.

### Luciferase Assays

The *Foxg1* overexpression plasmid (pCAG-*Foxg1*, NM_008241) was commercially synthesized from SYNBIO TECH company (Shenzhen, China). Reporter constructs containing FOXG1-binding regions from CUT&Tag analysis were generated by cloning genomic fragments (*Gria1*: chr11: 57,010,536–57,011,991; *Gria2*: chr3: 80,802,407–80,804,635; *Gria3*: chrX: 41,399,187–41,401,240) into promoter Glo 3 (pGL3-promoter) vector (primers in Table S3). All plasmids were sequence-verified.

Neuro2a cell line (CVCL_0470) was grown in complete Dulbecco’s Modified Eagle Medium (DMEM)/F12 medium (10% fetal bovine serum + antibiotics) under standard conditions. For transfection, 1.0 × 10^5^ cells were plated and transfected using Lipofectamine 2000 with: (1) 300 ng pCAG (control) or pCAG-*Foxg1*; (2) 300 ng reporter plasmid; (3) 30 ng plasmid Renilla luciferase (pRL)-SV40 (internal control). Following 24 h post-transfection incubation, cells were harvested and analyzed using Promega’s Dual-Luciferase system. Relative promoter activity was calculated as Firefly/Renilla ratio values (*n* = 3).

### Drug Administration

*Foxg1* cKO and control mice received daily subcutaneous injections of CX546 (Tocris #2980, 10 mg/kg in 1% dimethyl sulfoxide) or vehicle for 7 days, followed by behavioral or electrophysiological analyses. This dosage regimen was selected based on established protocols demonstrating that chronic AMPAR activation improves social deficits in ASD models [41, 42].

### Statistical Analyses

Data were expressed as the mean ± SEM and analyzed using appropriate statistical tests, including Unpaired *t*-test, Two-way repeated measures analysis of variance (ANOVA) followed by Slidák’s multiple comparisons test, Kolmogorov–Smirnov test, One-way ANOVA followed by Tukey’s multiple comparisons test, and hypergeometric test. Specific test details, sample sizes, and data presentation formats were provided in figure legends. Statistical thresholds were set at *P <0.05, **P <0.01, ***P <0.001.

## Results

### Deletion of *Foxg1* in iSPNs Results in ASD-like Behaviors in Mice

**Fig. 1:**
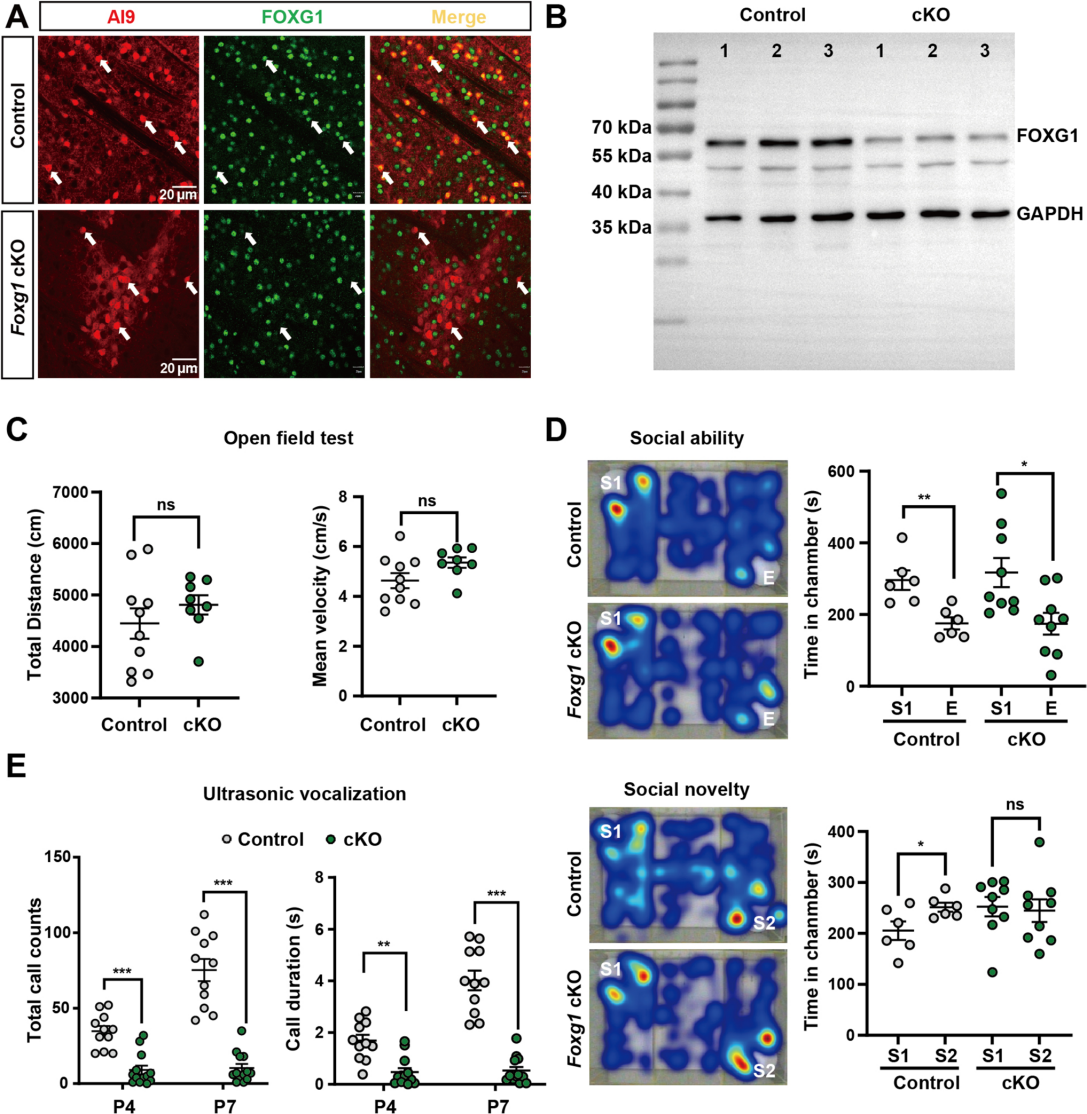
Conditional *Foxg1* knockout in iSPNs recapitulates ASD-like impairments in social novelty preference and communicative deficits in mice. **A** Immunostaining at P60 shows efficient FOXG1 knockout in AI9-labeled iSPNs (red). Scale bars, 20 μm. **B** Western blot analysis of P60 striatal extracts verifies FOXG1 disruption. **C** Open field test reveals comparable total distance traveled (left) and average velocity (right) >30 min between genotypes (Control: *n* = 10; *Foxg1* cKO: *n* = 8). **D** Three chamber test demonstrates intact social ability (Upper, empty *vs* Stranger 1) but impaired social novelty preference (Lower, Stranger 2 *vs* Stranger 1) in *Foxg1* cKO mice (Control: *n* = 6; *Foxg1* cKO: *n* = 9). Colors correspond to the strength of social preference, from low (blue) to high (red). **E** Neonatal ultrasonic vocalization (USV) test at P4 and P7 detects fewer total call counts (left) and shorter call duration (right) in *Foxg1* cKO pups (Control: *n* = 11; *Foxg1* cKO: *n* = 13). Data are presented as the mean ± SEM. Unpaired *t*-test for panels **C**, **D**, and **E**. For all the panels, dots represent individual mice. *P <0.05, **P <0.01, ***P <0.001; ns, not significant. E, empty; S1, stranger 1; S2, stranger 2; cKO, conditional knockout.

To explore the role of FOXG1 in iSPNs, we specifically deleted *Foxg1* using *Drd2-Cre* with an *Ai9* line introduced as a reporter to label iSPNs (*Drd2-Cre;Foxg1*^fl/fl^*;Ai9,* hereafter referred to as *Foxg1* cKO) (Fig. 1A, B). The body weight and the brain size of *Foxg1* cKO mice were comparable to their control littermates (*Foxg1*^fl/fl^*;Ai9,* hereafter referred to as control mice). In the open field test, both genotypes displayed similar levels of the total distance traveled and average velocity within 30 min (Fig. 1C), indicating the spontaneous locomotor activity was not affected after *Foxg1* deletion. A three-chamber test showed that both genotypes spent a longer duration of time in the chamber containing a stranger 1 mouse than in an empty chamber (Fig. 1D), demonstrating a normal social ability in *Foxg1* cKO mice. However, compared to a longer duration of time in the chamber containing an unfamiliar mouse stranger 2 than that in the familiar stranger 1 in control mice, *Foxg1* cKO mice did not show a preference for the stranger 2 (Fig. 1D), implying that the social novelty period was significantly impaired. Individuals with ASD frequently encounter challenges in language communication, especially in the adolescent phase [43–45]. As a key component of murine social behavior, ultrasonic vocalizations serve as an important communication channel that is perceptible only within their species. We thus assessed the communicative behavior of pups toward the mother by the neonatal ultrasonic vocalization (USV) test at P4 and P7. Our results revealed that *Foxg1* cKO pups emitted fewer total call counts and call durations than control mice (Fig. 1E), suggesting a communication dysfunction during early life in *Foxg1* cKO mice.

**Fig. 2:**
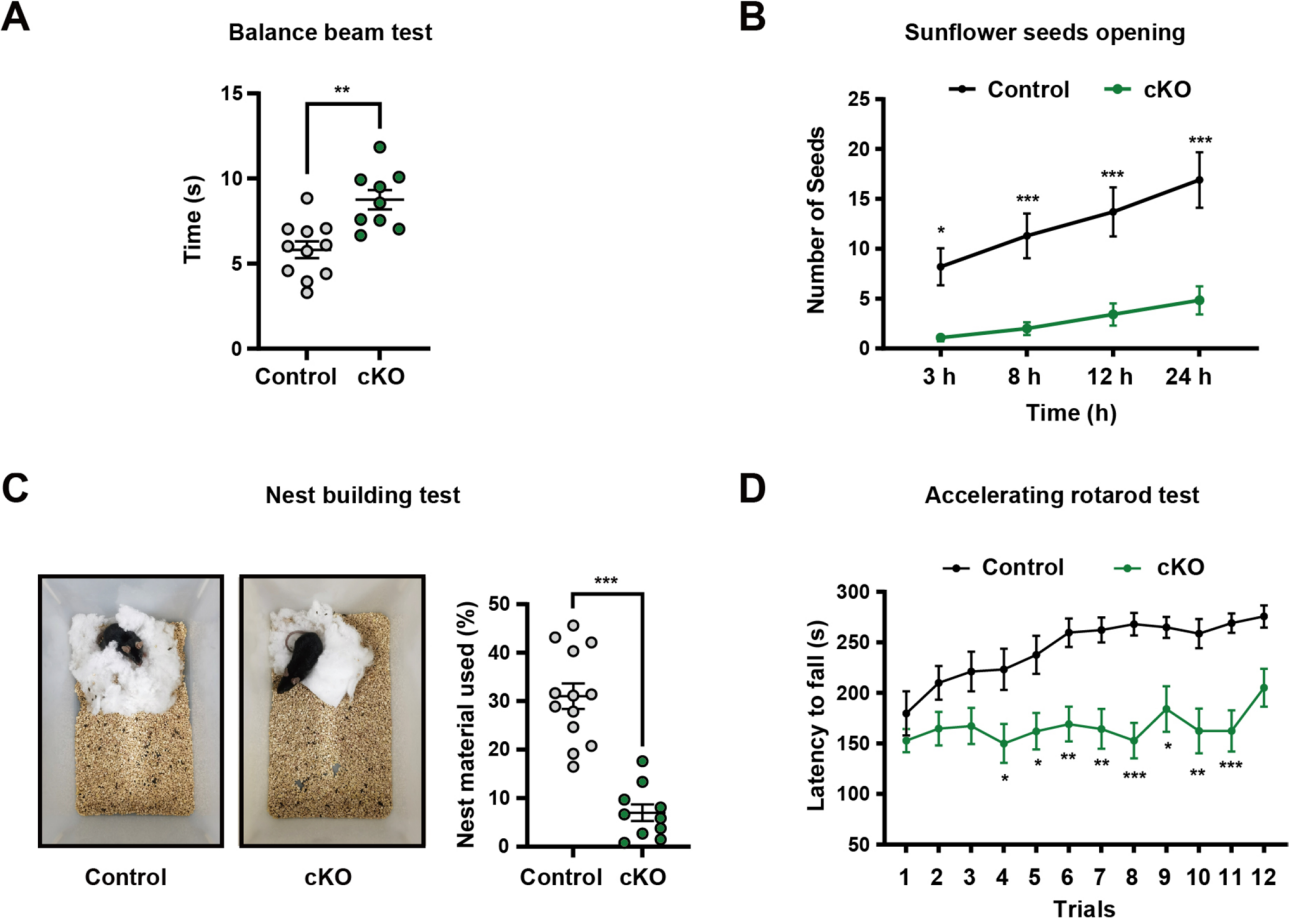
Conditional *Foxg1* knockout in iSPNs disrupts fine motor skill in mice. **A–C** Fine movement assessments in *Foxg1* cKO mice identify deficits with prolonged balance beam traversal time (**A**, Control: *n* = 11; *Foxg1* cKO: *n* = 9), reduced number of sunflower seeds successfully peeled (**B**, Control: *n* = 10; *Foxg1* cKO: *n* = 12), and poorer nest building quality (**C**, Control: *n* = 13; *Foxg1* cKO: *n* = 10) when compared to controls.** D** Accelerating rotarod test indicates motor learning impairment (Control: *n* = 13; *Foxg1* cKO: *n* = 17). Data are presented as the mean ± SEM. Unpaired *t*-test for panels **A** and **C**; two-way repeated measures ANOVA followed by Slidák’s multiple comparisons test for panels **B** and **D**. For all the panels, dots represent individual mice. *P <0.05, **P <0.01, ***P <0.001. cKO, conditional knockout.

Since ASD patients often exhibit comorbidities such as impaired fine movement, we designed three behavioral paradigms, balance beam performance, sunflower seeds opening task, and nest building test to evaluate fine motor skills. *Foxg1* cKO mice displayed pronounced deficits in fine motor skills across all these three behavioral assays compared to control mice, notably as evidenced by longer time to cross the balance beam (Fig. 2A), fewer number of opening sunflower seeds (Fig. 2B) and worse nesting performance (Fig. 2C). We also performed the accelerated rotarod test and detected a decreased motor learning ability in *Foxg1* cKO mice (Fig. 2D). Taken together, loss of *Foxg1* in striatal iSPNs leads to typical ASD-like behaviors, including impaired social novelty and communication difficulties, and ASD-associated abnormalities, such as fine movement impairments and decreased motor learning abilities.

### Loss of *Foxg1* Leads to Abnormal Accumulation of iSPNs and Decreased Dendritic Complexity and Spine Density

**Fig. 3:**
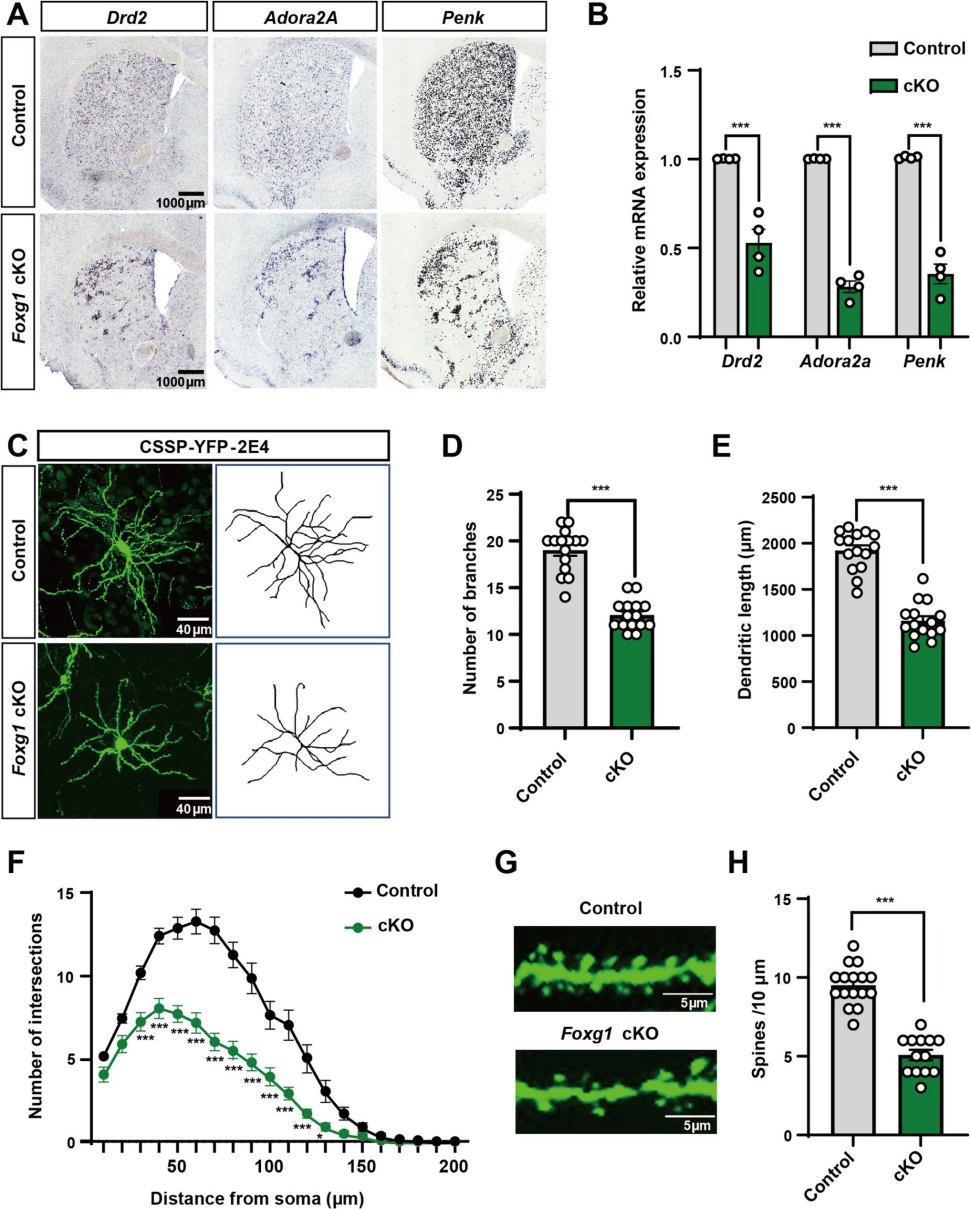
*Foxg1* deletion in iSPNs disrupts neuronal distribution and dendritic morphology. **A** *In situ* hybridization for iSPNs markers (*Drd2*, *Adora2a*, *Penk*) in striatal sections demonstrates reduced number and abnormal clustering of iSPNs in *Foxg1* cKO mice. Scale bars, 1000 μm. **B** Quantitative PCR analyses reveal significant down-regulation of striatal mRNA levels in *Foxg1* cKO mice, with reductions of 47.11% in *Drd2*, 71.90% in *Adora2a*, and 64.90% in *Penk* compared to controls (*n* = 4 biological replicates). **C** Representative images of viral-labeled iSPNs in P60 striatum. Scale bars, 40 μm. **D, E** *Foxg1* cKO iSPNs exhibit fewer dendritic branches and shorter dendritic length. **F** Sholl analyses indicate diminished dendritic complexity of *Foxg1* cKO iSPNs. **G** Representative images of spines from secondary branches. Scale bars, 5 μm.** H** Spine density is markedly lower in *Foxg1* cKO iSPNs. Sample sizes for panels **D**, **E**, **F**, and **H**: *n* = 15 neurons from 3–4 mice/group. Data are presented as the mean ± SEM. Unpaired *t*-test for panels **B**, **D**, **E**, and **H**, two-way repeated measures ANOVA followed by Slidák’s multiple comparisons test for panel **F**. *P <0.05, ***P <0.001. cKO, conditional knockout.

To investigate the impact of *Foxg1* deletion on the development of iSPNs, we performed *in situ* hybridization of *Drd2* and *Adora2a*, two specific markers commonly used for iSPNs. We detected a significant reduction in the number of iSPNs in the *Foxg1* cKO striatum, and this observation was further corroborated by staining for *Penk*, a marker for iSPNs that is positioned in the striatal matrix compartment (Fig. 3A). qPCR analyses consistently demonstrated a substantial decline in mRNA levels of *Drd2*, *Adora2a*, and *Penk*, with decreases of 47.11%, 71.90%, and 64.90%, respectively, in the *Foxg1* cKO striatum when compared to controls (Fig. 3B). Intriguingly, we observed remarkable accumulated clusters of iSPNs in the *Foxg1* cKO striatum (Fig. 3A), indicating that FOXG1 is required for the proper positioning of iSPNs. Recently, studies have shown that mutations in ASD risk genes, such as *Nlgn1*, *Nlgn3*, and *Cntnap2*, result in cellular migration and adhesion defects accompanied by social abnormalities and language communication deficits [46–49], suggesting a potential causal role of cellular migration and adhesion processes in ASD. Thus, the reduced number and the abnormal accumulation of iSPNs may contribute, at least in part, to the ASD-like behaviors observed in our *Foxg1* cKO mice.

Developmental abnormalities in dendrite arborization and spine density are closely linked to ASD, as evidenced by postmortem analyses of ASD human brains and animal models [50–53]. To assess morphological changes in *Foxg1* cKO iSPNs, we sparsely labelled iSPNs utilizing a Cre-dependent viral labeling strategy at P60 (Fig. 3C). Quantitative analyses revealed that *Foxg1* cKO iSPNs exhibited fewer dendritic branches and an obviously reduced total length of dendrites (Fig. 3D, E). Dendritic complexity was quantified by counting intersections between dendrites and concentric circles (10 μm spacing) radiating from the soma. Sholl analysis showed a significantly decreased number of intersections in *Foxg1* cKO iSPNs, further supporting a simplified dendritic arborization (Fig. 3F). We also observed a remarkable reduction in dendritic spine density in *Foxg1* cKO iSPNs compared to that in the control mice (Fig. 3G, H). Collectively, these results suggest that *Foxg1* deletion in iSPNs leads to developmental defects, spanning from aberrant distribution to diminished dendritic complexity and spine density.

### AMPAR-Mediated Excitatory Synaptic Transmission Is Attenuated After *Foxg1* Deletion

**Fig. 4:**
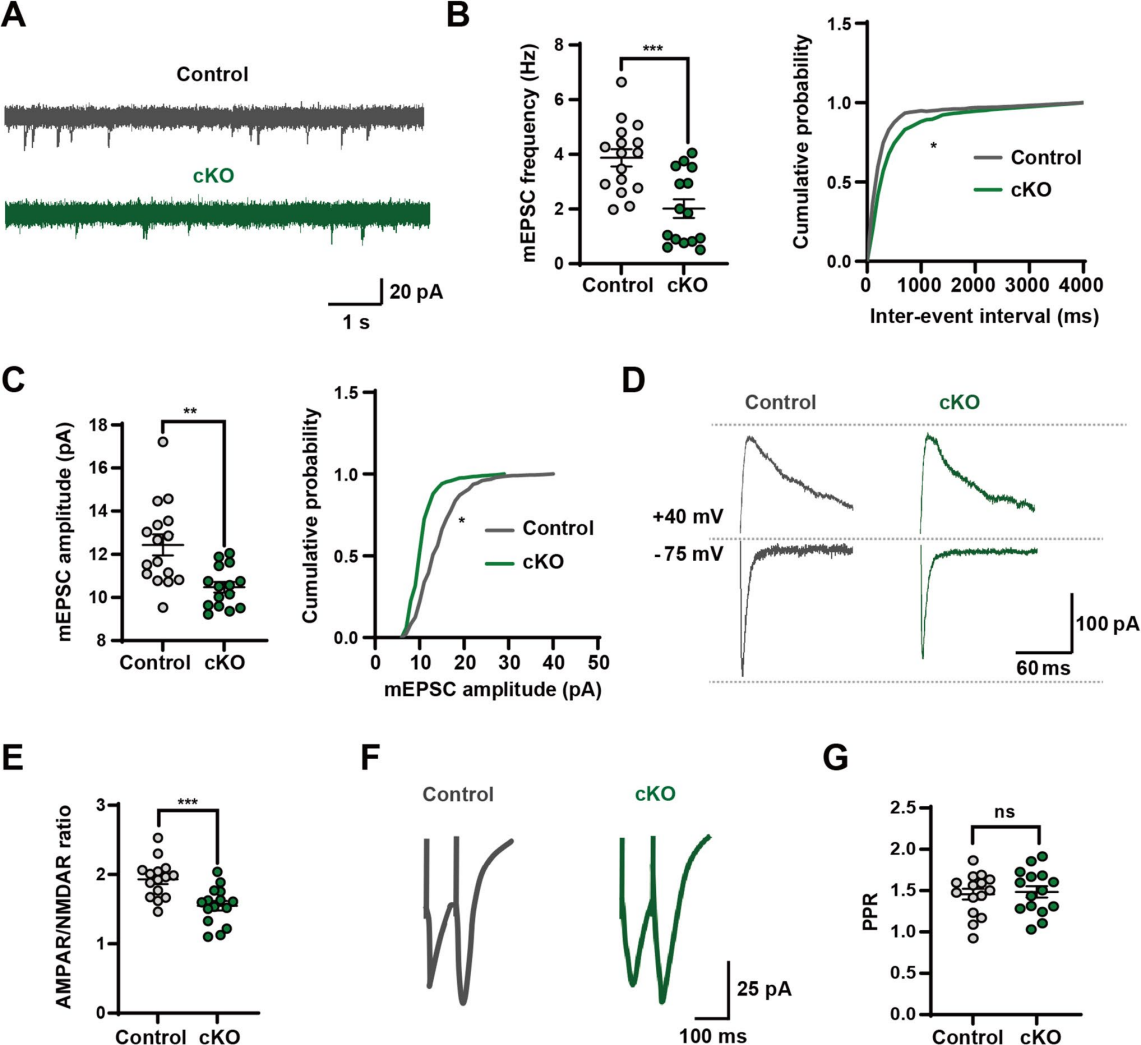
*Foxg1* deletion impairs AMPAR-mediated excitatory synaptic transmission in iSPNs. **A** Representative traces of mEPSCs recorded in *Foxg1* cKO and control iSPNs. Scale bar, 1 s/20 pA. **B, C** Statistical graph of mEPSCs frequency and peak amplitude, showing a significant reduction in *Foxg1* cKO iSPNs compared to controls (Control: *n* = 16 neurons from 3 to 4 mice; *Foxg1* cKO: *n* = 15 neurons from 3 to 4 mice). **D** Sample traces of AMPAR/NMDAR ratio in *Foxg1* cKO and control iSPNs. Scale bar, 60 ms/100 pA. **E** Quantification analyses demonstrate that the AMPAR/NMDAR ratio is markedly decreased in *Foxg1* cKO iSPNs (Control: *n* = 15 neurons from 3–4 mice; *Foxg1* cKO: *n* = 15 neurons from 3 to 4 mice). **F** Representative traces of paired-pulse evoked EPSCs ratio (PPR) in iSPNs show comparable responses between *Foxg1* cKO and control groups. **G** Quantitative analyses confirm no significant difference in PPR between genotypes (Control: *n* = 15 neurons from 3 to 4 mice; *Foxg1* cKO: *n* = 15 neurons from 3 to 4 mice). Data are presented as the mean ± SEM. Statistical significance was assessed using an unpaired two-tailed *t*-test for all comparisons, except for cumulative probability analyses, which employed the Kolmogorov–Smirnov test. *P <0.05, **P <0.01, ***P <0.001; ns, not significant. cKO, conditional knockout.

To examine the functional consequences of *Foxg1* deletion in iSPNs, electrophysiological recordings were conducted in acute brain sections from P45–P60 mice. (Fig. S1A) and detected a modest reduction in the frequency of mEPSCs in *Foxg1* cKO iSPNs (Fig. 4A). Quantitatively, the mean mEPSCs frequency in *Foxg1* cKO iSPNs was 2.015 ± 0.328 Hz, representing a 45.35% decrease relative to the control value of 3.687 ± 0.309 Hz (Fig. 4B). Consistently, the cumulative probability distribution of inter-event intervals (IEIs) was right-shifted in *Foxg1* cKO iSPNs, supporting the decline in the occurrence of spontaneous synaptic events (Fig. 4B). We also observed a pronounced reduction in the peak amplitude of mEPSCs in *Foxg1* cKO iSPNs (Fig. 4A). The mean mEPSCs amplitude was 10.410 ± 0.236 pA, notably lower than that in control iSPNs (12.150 ± 0.472 pA), reflecting a 14.32% reduction (Fig. 4C). This was further corroborated by a left-shift in the cumulative probability distribution of mEPSCs amplitude, indicating a weakness in synaptic strength (Fig. 4C). Together, the concurrent changes in both mEPSCs frequency and amplitude upon *Foxg1* deletion in iSPNs underscore the role of FOXG1 in maintaining excitatory synaptic transmission, with its loss potentially contributing to the synaptic and behavioral deficits observed in *Foxg1* cKO mice.

The decrease in mEPSC amplitude often correlates with an impaired postsynaptic receptor function, potentially attributed to a diminution in the number or efficacy of functional postsynaptic receptors. Given the central role of AMPAR as the primary postsynaptic mediators of fast excitatory synaptic transmission [54, 55], we thus evaluated the AMPAR/NMDAR ratio, an indicator of synaptic strength and receptor composition. *Foxg1* cKO iSPNs exhibited a significantly reduced AMPAR/NMDAR ratio compared to controls (Fig. 4D, E), indicating AMPAR-mediated excitatory synaptic transmission was impaired. Since the reduction in mEPSC frequency, on the other hand, reflects a possible decrease in presynaptic release, to test this possibility, we examined the paired-pulse ratio (PPR), a well-established metric for assessing presynaptic release probability. Notably, no obvious differences in PPR were detected between *Foxg1* cKO and control iSPNs (Fig. 4F, G), effectively excluding presynaptic glutamate release differences as a major contributing factor. These findings reveal that the synaptic deficits in *Foxg1* cKO mice are likely attributable to postsynaptic mechanisms rather than presynaptic alterations.

Beyond synaptic transmission, intrinsic membrane properties critically shape neuronal firing patterns and functional output. To determine whether the observed excitatory synaptic transmission deficits might be secondary to the altered intrinsic neuronal excitability, we therefore characterized the intrinsic electrophysiological properties of iSPNs in *Foxg1* cKO mice. No significant differences were observed in resting membrane potential (RMP) or input resistance between genotypes (Fig. S1B, C), indicating that intrinsic membrane properties remained intact. The number of action potentials elicited by varying stimulus intensities was also comparable between genotypes (Fig. S1D, E). In summary, these findings demonstrate that *Foxg1* deletion specifically impairs AMPAR-mediated excitatory synaptic transmission without affecting intrinsic membrane properties in iSPNs, providing a potential postsynaptic mechanism for the ASD-like behavioral deficits observed in *Foxg1* cKO mice.

### FOXG1-driven Transcriptional Networks Hierarchically Shape Synaptic Functions of iSPNs Contributing to ASD Etiology

**Fig. 5:**
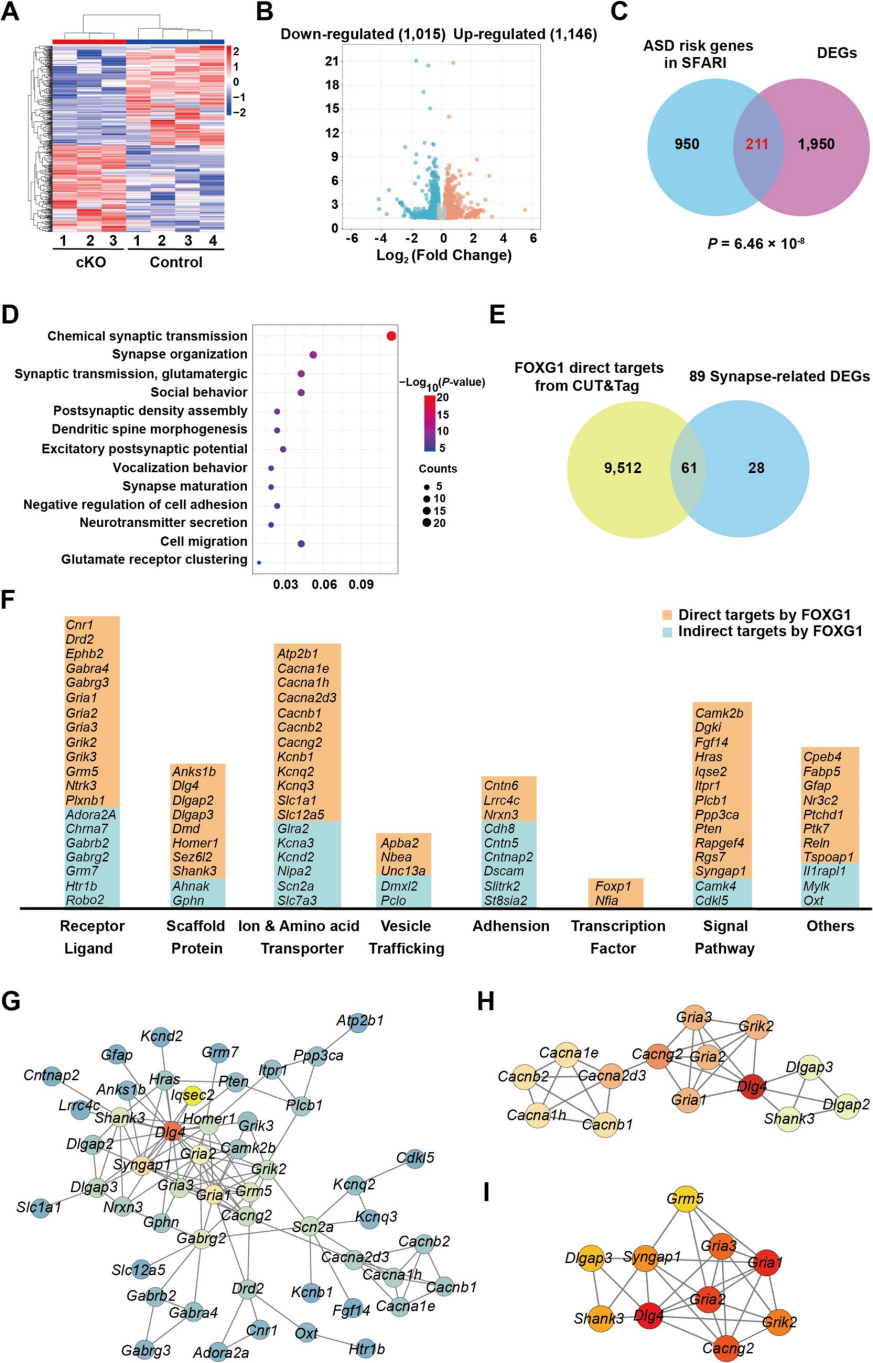
FOXG1 orchestrates transcriptional network regulating synaptic functions. **A** Heatmap of deregulated genes upon *Foxg1* mutation across all replicates, scaled by gene (rows). **B** Volcano plot of differential gene expression (DEGs) analysis by bulk RNA-seq of striatal samples from P30 *Foxg1* cKO and control mice. The red and blue circles respectively indicate 1,146 up-regulated and 1,015 down-regulated DEGs with *P-*value <0.05, absolute fold change >1.13. **C** Overlap between DEGs and ASD risk genes from the SFARI database, identifying 211 *Foxg1*-regulated ASD risk DEGs. The significance of the observed numbers of overlapping genes was calculated by hypergeometric tests.** D** Gene ontology (GO) analysis of biological processes enriched in 211 *Foxg1*-regulated ASD risk genes. Significantly enriched GO terms were selected as those with a *P-*value <0.05. **E** Overlap between FOXG1 direct target genes identified by CUT&Tag and 89 DEGs enriched in synapse-related GO terms.** F** Systematic identification of 89 FOXG1-regulated synaptic DEGs reveals multi-layered regulation of synaptic functions. Direct FOXG1 target genes (*n* = 61, identified by CUT&Tag) are highlighted with orange overlay, while indirectly regulated genes are shown in teal. **G** The protein-protein interaction network, constructed from proteins encoded by 89 synaptic-related genes using the STRING database (high confidence: 0.700), demonstrates significant interconnectivity (*P-*value = 1.0 × 10^−16^). Nodes represent genes and edges indicate functional associations. **H** Highest-scoring subnetwork identified by the Molecular Complex Detection (MCODE) algorithm (score = 4.923). **I** The top 10 hub genes identified by the CytoHubba algorithm. cKO, conditional knockout; DEGs, differentially expressed genes.

To elucidate the transcriptional mechanisms underlying iSPN function and ASD core symptoms, we performed bulk RNA sequencing (RNA-Seq) on striatal samples at P30 and identified 2161 differentially expressed genes (DEGs), with 1,146 up-regulated and 1,015 down-regulated (*P-*value <0.05, absolute fold change >1.13) (Fig. 5A, B). We then compared DEGs with annotated ASD risk genes from the Simons Foundation Autism Research Initiative Gene Database (SFARI) database, and finally identified 211 FOXG1-downstream ASD risk DEGs, underscoring a strong association between FOXG1-driven transcriptional networks and ASD susceptibility (Fig. 5C). GO analyses supported roles for these 211 DEGs mainly associated with terms of “dendritic spine morphogenesis”, “synaptic organization”, “synaptic maturation”, “ion transmembrane transport”, “glutamate receptor clustering”, “neurotransmitter secretion” as well as “chemical synaptic transmission”, emphasizing that FOXG1 hierarchically shapes synaptic functions in iSPNs (Fig. 5D). These synaptic-related categories encompassed 89 DEGs in which 61 were FOXG1-direct targeted genes identified by CUT&Tag sequencing (Figs 5E and S2). Noteworthy, terms such as “cell migration” and “cell adhesion” were also identified (Fig. 5D), consistent with the accumulation of iSPNs in our *Foxg1* cKO mice.

The roles of 61 FOXG1-direct targeted genes were involved in receptors including *Cnr1*, and the core AMPAR subunit *Gria1*, *Gria2*, and *Gria3*; scaffolding proteins including *Dlg4* and *Shank3*; ion transporters including Ca^2+^ and K^+^ channel family members; vesicle trafficking proteins *Apba2* and *Nbea*; cell adhesion molecules including *Cntn6*, *Nrxn3*; transcriptional factors including *Foxp1*, *Nifa* and proteins that involved in signal transduction such as *Camk2b*, *Pten*, and *Syngap1* (Fig. 5F). The cannabinoid receptor gene *Cnr1*, abundantly localized to iSPN axonal terminals and collaterals, is known to modulate emotional and stress-related responses [56–58]. Here, we found that *Cnr1* was incorporated into the gene networks directly regulated by *Foxg1* to orchestrate striatal output (Fig. S3). The significant down-regulation of a well-known encoding postsynaptic scaffolding protein ASD risk gene *Shank3* in *Foxg1* cKO mice is particularly noteworthy (Fig. S3), given that *Shank3* loss-of-function in iSPNs is known to decrease excitatory synaptic transmission frequency/amplitude and disrupt synaptic plasticity [26, 59]. *Syngap1* encodes a GTPase-activating protein, and its haploinsufficiency in iSPNs has been reported to impair excitatory synaptic transmission and alter goal-directed behavior [60]. In our RNA-Seq study, *Syngap1* was down-regulated in *Foxg1* cKO mice, indicating that *Foxg1* regulates the *Syngap1*-mediated signaling pathway contributing to the function of iSPN circuits. It is worth mentioning that we also identified *Anks1b*, a structural organizer within the postsynaptic density [61, 62], and *Cntn6,* a synaptic adhesion molecule [63], as FOXG1 directly targeted genes with their mRNA expression levels down-regulated after *Foxg1* deletion (Fig. S3), suggesting that *Foxg1* is required for synapse architecture in iSPNs as well. Collectively, these findings delineate that FOXG1 functions as a hierarchical transcriptional coordinator of iSPN synaptic functions.

We next performed Protein-Protein Interaction analysis (PPI) using the STRING database and revealed significantly enriched interactions (*P-*value = 1.0 × 10^−16^) (Fig. 5G). By the Molecular Complex Detection algorithm (MCODE) in Cytoscape, we pinpointed sub-networks that capture the most robustly affected synaptic processes and identified a top-scoring module (score = 4.923) enriched in *Gria1*, *Gria2*, and *Gria3* that encode the core AMPAR subunit GluR1, GluR2, and GluR3, respectively (Fig. 5H). We also identified *Cacng2* in this sub-network (Fig. 5H), which encodes an AMPAR auxiliary subunit previously reported to enhance AMPAR activity [55, 64]. The reduced *Cacng2* mRNA levels after *Foxg1* deletion may contribute to the diminished AMPAR-mediated excitatory synaptic transmission observed in *Foxg1* cKO iSPNs (Fig. S3). Coherently, the down-regulation of scaffolding protein-encoding genes *Dlg4*, *Dlgap2*, and *Dlgap3*, known regulators of AMPAR trafficking, may account for the reduced AMPAR-mediated currents [16, 65–67] (Fig. S3). Moreover, using the Maximal Clique Centrality algorithm in CytoHubba, we validated the top 10 hub genes as central nodes within the PPI network, including the aforementioned AMPAR-related genes such as *Gria1*, *Gria2*, *Gria3*, *Cacng2*, *Dlg4*, and *Dlgap3* (Fig. 5I), further supporting the crucial role of these FOXG1-targeted AMPAR-related genes in iSPNs. Overall, our results establish that FOXG1 orchestrates a transcriptional network essential for synaptic function, with a particularly notable role in regulating AMPAR-mediated synaptic transmission.

### FOXG1 Directly Activates the Core AMPAR Subunit* Gria1, Gria2,* and *Gria3*

**Fig. 6:**
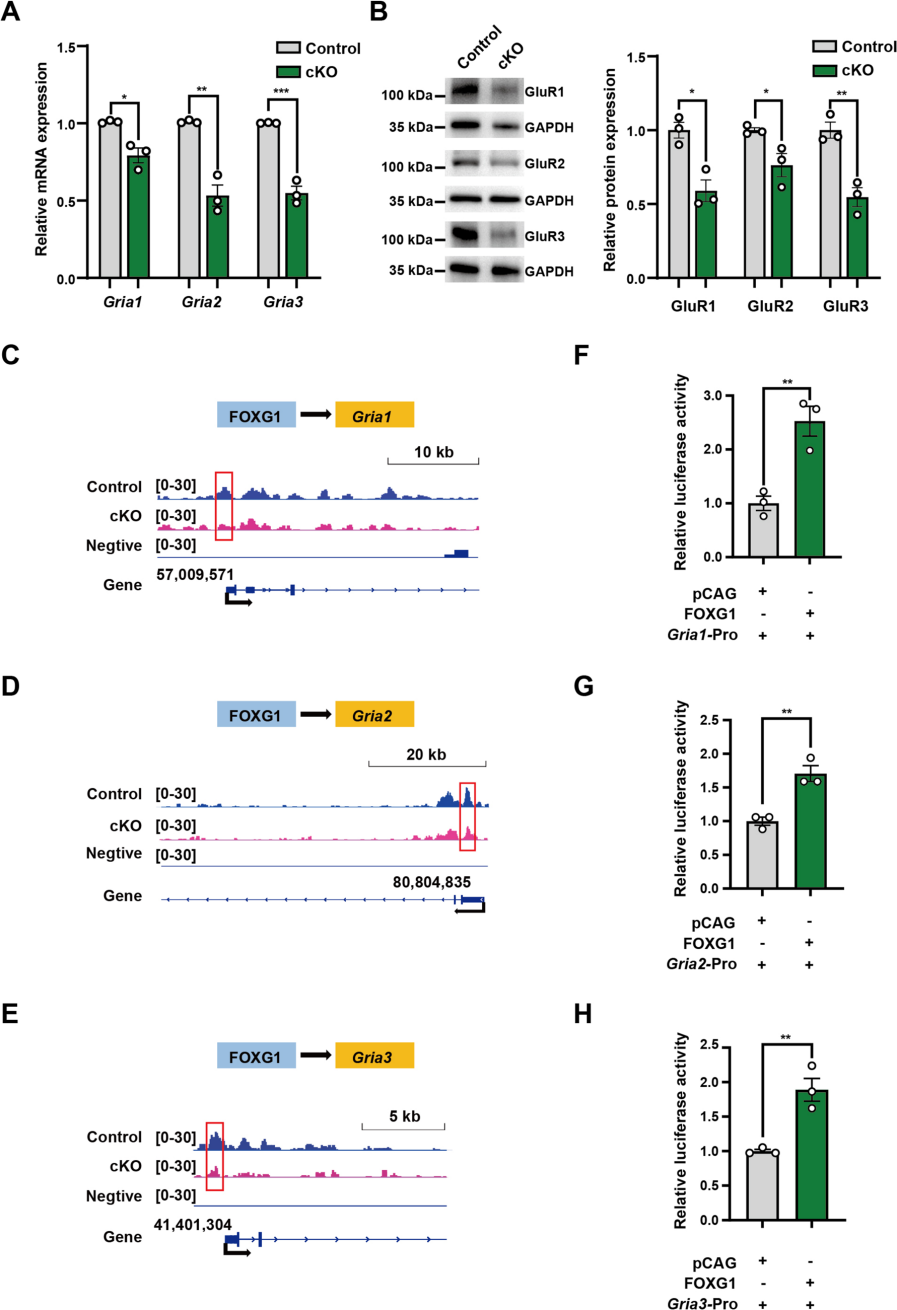
FOXG1 directly activates AMPAR subunit genes *Gria1*, *Gria2,* and *Gria3*. **A** Quantitative PCR analyses reveal significant down-regulation of striatal mRNA levels in *Foxg1* cKO mice, with reductions of 21.55% in *Gria1*, 47.20% in *Gria2*, and 45.20% in *Gria3* compared to controls (*n* = 3 biological replicates).** B** The protein levels of GluR1, GluR2, and GluR3 were examined by Western blot (*n* = 3 biological replicates). **C–E** Primary mouse striatal nuclear suspensions from *Foxg1* cKO and control mice were subjected to CUT&Tag-sequencing analyses using an anti-FOXG1 antibody. The sequencing results were used to quantify the abundance of *Gria1*, *Gria2*, and *Gria3* enriched by FOXG1. The distribution of FOXG1 binding at the promoter regions of *Gria1*, *Gria2*, *Gria3*, and their impact are shown in **C**, **D**, and **E**, respectively. Red boxes indicate the regions that are enriched for FOXG1 binding. **F–H** Luciferase assays showing that FOXG1 directly activates *Gria1*, *Gria2*, and *Gria3* (*n* = 3 biological replicates). Data are presented as the mean ± SEM. Statistical significance was assessed using an unpaired two-tailed *t*-test for all comparisons. *P <0.05, **P <0.01, ***P <0.001. cKO, conditional knockout.

Next, we aimed to examine the direct regulation of FOXG1 on three candidate genes, *Gria1*, *Gria2*, and *Gria3*. qPCR analyses demonstrated that mRNA levels of *Gria1*, *Gria2*, and *Gria3* decreased by 21.55%, 47.20%, and 45.20%, respectively in *Foxg1* cKO striatum (Fig. 6A), and Western blot tests confirmed a substantial reduction in protein levels of GluR1, GluR2, and GluR3 (Fig. 6B). Integrative Genomics Viewer (IGV) analyses of CUT&Tag further identified putative FOXG1-binding sites within the promoter regions of *Gria1*, *Gria2*, and *Gria3*, with a significant decrease in binding peaks upon *Foxg1* deletion (Fig. 6C, D, E). We cloned the putative promoter regions near the transcription start site (TSS) of *Gria1* (1,456 bp), *Gria2* (2,229 bp), and *Gria3* (2054 bp) into luciferase reporter plasmids and then transfected them into N2A cells together with the *Foxg1* expression plasmid, respectively. Luciferase assays showed that FOXG1 overexpression consistently enhanced luciferase activity across all three candidate genes, with an increase of 2.641-fold for *Gria1*, 1.707-fold for *Gria2*, and 1.743-fold for *Gria3* relative to the empty vector control (Fig. 6F, G, H). These findings establish that FOXG1 functions as a direct transcriptional activator of *Gria1*, *Gria2*, and *Gria3*, highlighting its central regulatory function in maintaining AMPAR-mediated synaptic transmission in iSPNs.

### Pharmacological Enhancement of AMPAR Function Rescues Synaptic Dysfunction and ASD-like Behaviors in *Foxg1* cKO Mice

**Fig. 7:**
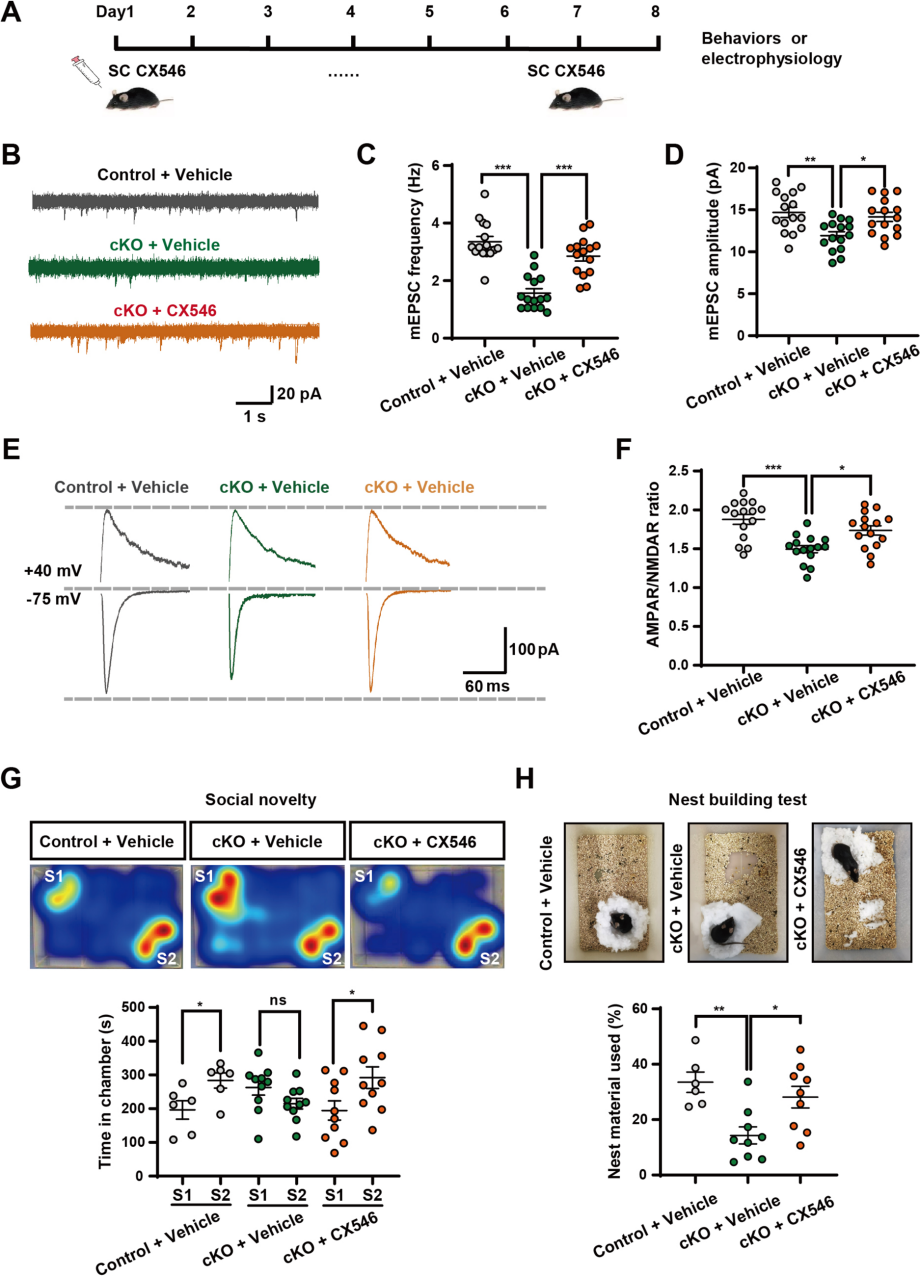
Systemic administration of an AMPAR-positive modulator CX546 improves synaptic and behavioral deficits in *Foxg1* cKO mice. **A** Adult *Foxg1* cKO and control mice receive daily subcutaneous injections of the AMPAR-positive allosteric modulator CX546 (10 mg/kg per day) for 7 consecutive days. Synaptic physiology and behavioral assays are performed on day 8 post-treatment. **B** Representative traces of mEPSCs recorded in control-vehicle, *Foxg1* cKO-vehicle, and *Foxg1* cKO-CX546 groups. Scale bar, 1 s/20 pA. **C, D** Statistical graph showing improvement in mEPSCs frequency and peak amplitude after administration of CX546 (*n* = 15 neurons from 3 to 4 mice per group). **E** Representative traces of AMPAR/NMDAR-mediated EPSCs recorded in the iSPNs in the three groups. Scale bar, 60 ms/100 pA. **F** The AMPAR/ NMDAR ratio is higher in the CX546-treated *Foxg1* cKO group (*n* = 15 neurons from 3 to 4 mice per group). **G** CX546 administration rescues social novelty preference (Control + Vehicle: *n* = 6; *Foxg1* cKO + Vehicle: *n* = 10; *Foxg1* cKO + CX546: *n* = 10) in CX546-treated *Foxg1* cKO mice. **H** CX546 administration improves nest building performance (Control + Vehicle: *n* = 6; *Foxg1* cKO + Vehicle: *n* = 9; *Foxg1* cKO + CX546: *n* = 9) in CX546-treated *Foxg1* cKO mice. Data are presented as the mean ± SEM. One-way ANOVA followed by Tukey’s multiple comparisons test for panels **C**, **D**, **F**, and **H**, unpaired two-tailed *t*-test for panel **G**. *P <0.05, **P <0.01, ***P <0.001; ns, not significant. cKO, conditional knockout.

To further investigate the regulation of FOXG1 in AMPAR-mediated excitatory synaptic transmission that contributes to ASD etiology, we conducted rescue experiments in *Foxg1* cKO mice by administering ampakine drug CX546, a positive allosteric modulator of AMPAR. On the eighth day post-administration, *Foxg1* cKO mice were subjected to measurements of synaptic activity and behavioral assessments (Fig. 7A). The frequency of mEPSCs in CX546-treated *Foxg1* cKO iSPNs increased to 2.826 ± 0.191 Hz, representing a 1.861-fold increase over vehicle-treated *Foxg1* cKO iSPNs (1.518 ± 0.144 Hz) (Fig. 7B, C). Concurrently, the amplitude of mEPSCs exhibited a robust enhancement, rising from 11.939 ± 0.446 pA in vehicle-treated *Foxg1* cKO iSPNs to 14.244 ± 0.553 pA in CX546-treated *Foxg1* cKO iSPNs, indicative of strengthened excitatory synaptic transmission after CX546 application (Fig. 7B, D). Notably, the AMPAR/NMDAR ratio was significantly higher in CX546-treated *Foxg1* cKO iSPNs (1.712 ± 0.062) compared to vehicle-treated *Foxg1* cKO iSPNs (1.504 ± 0.046), further supporting the specific potentiation of postsynaptic AMPAR responsiveness (Fig. 7E, F). Despite these postsynaptic improvements, the PPR remained unchanged between CX546-treated *Foxg1* cKO iSPNs and either vehicle-treated controls or vehicle-treated *Foxg1* cKO iSPNs (Fig. S4A). Overall, these findings demonstrate that CX546 effectively rescues AMPAR-mediated excitatory synaptic transmission deficits in *Foxg1* cKO mice, providing new insights into its therapeutic potential for synaptic dysfunction.

In terms of ASD-like behaviors, CX546-treated *Foxg1* cKO mice displayed a clear preference for interacting with a novel mouse over a familiar one in the social novelty period, in contrast to vehicle-treated *Foxg1* cKO mice, which showed no significant social novelty preference (Fig. 7G). This improvement suggests that CX546 rescues the social novelty deficits typically observed in ASD models. Interestingly, CX546-treatment normalized skillful movement deficits in the nest building test (Fig. 7H). Vehicle-treated *Foxg1* cKO mice utilized only 14.30% of the provided nesting material, notably less than control mice, which used 33.50% of the material. In contrast, CX546-treated *Foxg1* cKO mice used 28.15% of the material, a level comparable to controls, indicating that CX546 successfully restored fine movement in *Foxg1* cKO mice. Although CX546-treatment did not significantly rescue the capacity for sunflower seeds opening, a task assessing complex motor skills and coordination [39], an upward trend in improvement was observed (Fig. S4B). Taken together, our findings uncover that FOXG1 regulates AMPAR-mediated synaptic transmission in striatal iSPNs, contributing to ASD etiology, and suggest the potential of AMPAR activation as a therapeutic intervention for ASD and FOXG1 Syndrome.

## Discussion

It has been reported that the striatal indirect pathway is closely related to ASD [24, 26, 27, 68]. In this study, we took advantage of the *Drd2-Cre* line to disrupt *Foxg1*, a transcriptional factor required for telencephalic development, in postmitotic iSPNs. We found that *Foxg1* deletion in iSPNs led to ASD-like behaviors accompanied by reduced dendritic complexity, spine density, and impaired AMPAR-mediated synaptic transmission. We uncovered that FOXG1 functions as a hierarchical transcriptional coordinator of iSPN synaptic organization and function, whose dysfunction precipitates multisystem failures spanning from spine morphogenesis, synaptic organization, synaptic maturation, to ion transport, glutamate receptor clustering, neurotransmitter secretion, and synaptic transmission. We identified AMPAR subunit *Gria1*, *Gria2,* and *Gria3* as hub genes among the FOXG1-driven transcriptional network that governs synaptic functions in iSPNs. More importantly, an AMPAR-positive modulator, CX546, was effective for restoring the synaptic transmission deficits and ASD-like behaviors in *Foxg1* cKO mice. Our study uncovers a novel role of *Foxg1* in iSPNs, deepens the understanding of ASD etiology, and offers new insights into therapeutic strategy.

Synapse formation and strengthening require precisely regulated intracellular signaling and coordinated pre- and post-synaptic activity [69–72]. However, the transcription factors that orchestrate the synaptic functions are relatively underexplored. Here, we presented the systematic identification of synaptic regulators under the control of FOXG1 in iSPNs. Our analyses revealed functionally interconnected modules that include synaptic receptors, scaffolding proteins, transporters, vesicle trafficking related molecules, adhesion molecules, and transcriptional/signaling regulators. For example, one of our findings was that FOXG1 directly activates the cannabinoid receptor gene *Cnr1* in iSPNs. *Cnr1* has been reported to regulate neural function by balancing excitatory and inhibitory synaptic transmission, and selective ablation of cannabinoid receptors in striatal spiny projection neurons (SPNs) leads to exploration behavior deficits and impaired motor coordination in mice [56–58]. Among genes that encode scaffolding proteins, in addition to the well-known ASD risk genes *Shank3* and *Dlg4*, we identified *Anks1b*, a postsynaptic density organizer that forms stable complexes with post-synaptic density protein 95 (PSD-95) to maintain excitatory synapse architecture, as a FOXG1-targeted gene. Ankyrin repeat and SAM domain-containing 1B (ANKS1B) function likely underlies the strong genetic association between ANKS1B variants and neurodevelopmental disorders, including ASD, attention-deficit/hyperactivity disorder (ADHD), and speech disorders [61, 62, 73]. We also identified a set of ion transporters (*Cacna1e*, *Cacna1h*, *Cacnb1*, *Cacnb2*, and *Kcnq2*, *Kcnq3*, *Kcnb1*), as well as vesicle trafficking molecules (*Apba2*, *Nbea*, *Unc13a*), and synaptic adhesion molecules (*Cntn6*, *Lrrc4c*), that are driven by FOXG1 in iSPNs. These findings establish that FOXG1 acts as a hierarchical transcriptional coordinator in synaptic organization and function in iSPNs and thus expand the understanding of the mechanisms underlying iSPNs development and related disorders.

Studies have shown dysfunction of AMPAR, such as distinct changes in AMPAR compositions and AMPAR/NMDAR ratio in ASD [74, 75]. Here, we uncovered FOXG1-driven transcriptional program governing excitatory synaptic transmission, pinpointing AMPAR hypofunction in iSPNs as the most salient feature correlating with both synaptic and behavioral deficits reminiscent of ASD. We found a convergent suppression of AMPAR function-related transcripts, including *Gria1*, *Gria2*, *Gria3*, *Cacng2*, *Dlg4*, *Dlgap2*, and *Dlgap3*, which are in line with the reduction in AMPAR-mediated currents in *Foxg1* cKO mice. *Gria1*, *Gria2,* and *Gria3* are known to encode the core AMPAR subunits. We demonstrated that FOXG1 directly binds to the promoter regions and transcriptionally activates *Gria1*, *Gria2,* and *Gria3* genes. It is worth mentioning that we also identified coordinated down-regulation of AMPAR regulatory components such as *Cacng2* in *Foxg1* cKO mice. *Cacng2* encodes the transmembrane AMPA receptor regulatory protein γ2 (TARP-γ2) auxiliary subunit Ca^2+^ channel gamma subunit 2 (CACNG2) that enhances AMPAR function through coordinated interactions with the core AMPAR subunits, where it modulates AMPAR gating properties while promoting receptor trafficking to both the plasma membrane and synaptic sites [54, 64, 76]. Furthermore, we identified that FOXG1 activates *Dlg4*, *Dlgap2*, and *Dlgap3*, three scaffolding proteins that have been previously reported to be crucial for AMPAR-mediated synaptic transmission [16, 65–67]. Down-regulation of the three genes likely contributed to the attenuation of AMPAR-mediated currents in *Foxg1*-deficient iSPNs. Thus, our findings provide new insights into the regulation of AMPAR-mediated synaptic transmission.

In the present study, treatment with CX546, an AMPAR-positive allosteric modulator, effectively restored excitatory synaptic transmission and improved social novelty as well as nesting behaviors in *Foxg1* cKO mice, indicating that proper AMPAR-mediated excitatory synaptic transmission is essential for these behaviors. The successful rescue provides a promising therapeutic implication for ASD. Future studies focusing on AMPAR and its modulators as a potential therapeutic strategy would be particularly interesting. However, CX546 failed to fully restore fine movement performance in the sunflower seeds opening test, suggesting that AMPAR potentiation alone is insufficient to compensate for all behavioral deficits. This incomplete rescue likely stems from impairments in other FOXG1-regulated synaptic mechanisms beyond AMPAR. Other non-AMPAR synaptic genes downstream of FOXG1 (e.g., *Anks1b*, *Shank3*) are also key drivers of ASD-like behaviors, with non-negligible roles.

Studies using ASD human brains and animal models have revealed developmental abnormalities in synaptogenesis [50–53]. Here, we identified *Nrxn3* as directly regulated by FOXG1. *Nrxn3*, which is known to regulate synaptogenesis through interactions with Neuroligin 3. Disruption of this interaction has been demonstrated to cause social and motor learning deficits in mouse models [48, 77]. In DEGs after *Foxg1* deletion, we also identified high-risk ASD gene *Cntnap2*, which encodes a neurexin-family protein localized to the postsynaptic region to regulate synaptogenesis. *Cntnap2*-deficient mice recapitulate core ASD symptoms [46, 78]. Thus, both AMPAR-related genes and other synaptic genes regulated by FOXG1, such as *Anks1b*, *Shank3*, *Nrxn3*, and *Cntnap2*, contribute to the eventual ASD-like behavioral phenotypes. Future studies investigating the cooperative roles of these molecules in iSPNs would provide important insights into striatal development and ASD pathogenesis.

Previous studies have reported abnormal migration and mislocalization of cortical pyramidal neurons, and interneurons also contribute to ASD-like behaviors [46, 79, 80]. In our mouse model, *Foxg1* deletion resulted in aberrant clustering and disorganized cellular arrangement of iSPNs. Although the iSPNs-specific misorganization observed here has not been previously directly linked to ASD, it is plausible that aberrant striatal microcircuitry impairs the integrity of the indirect pathway, thereby contributing to behavioral impairments. Future studies that employ *in vivo* electrophysiology, fiber photometry, or optogenetics/chemogenetics to monitor and modulate the activity in downstream nuclei of the indirect pathway (e.g., the globus pallidus externa and subthalamic nucleus) would be interesting. Particularly, subsequent works that evaluate the therapeutic potential of CX546 in other ASD models and explore sex differences would provide new insights into therapeutic intervention for ASD.

## Supplementary Information

Below is the link to the electronic supplementary material.

Supplementary file1 (PDF 609 KB)

## Data Availability

The data that support the findings of this study are available from the corresponding author upon reasonable request.
