# Supplementary material for: FOXG1 Hierarchically Shapes Synaptic Functions in Striatal iSPNs and Contributes to ASD Etiology
Source: Neurosci Bull. 2026 Feb 2;42(5):1059–78. doi: 10.1007/s12264-025-01573-3 (PMC13158314; doi:10.1007/s12264-025-01573-3)
Supplement: Supplementary file 1 — Supplementary file1 (PDF 609 KB) [file 12264_2025_1573_MOESM1_ESM.pdf]

Supplementary Materials

Supplementary Figures

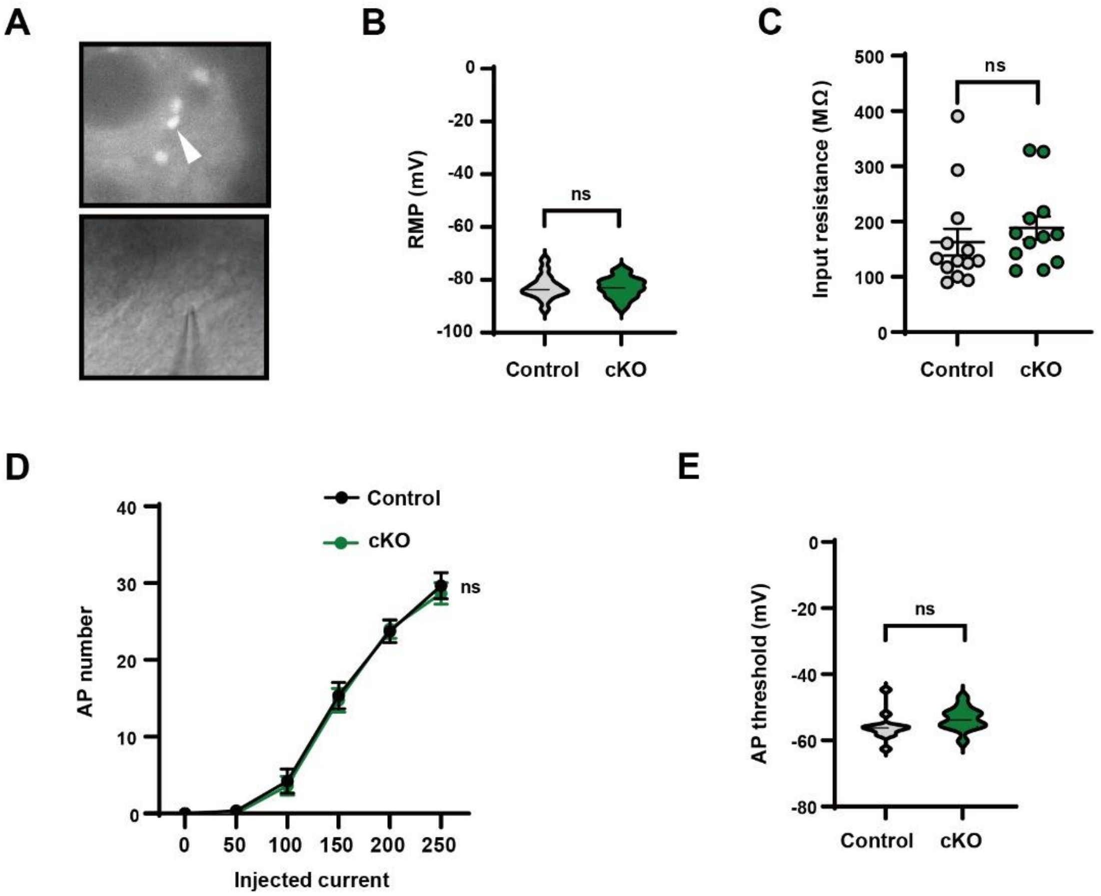

Fig. S1 Intrinsic electrophysiological properties of *Foxg1* cKO and control iSPNs. **A** Schematic of whole-cell patch-clamp recording in acute striatal slices from P45–P60 mice. White solid arrows indicate the recorded iSPNs. **B, C** Resting membrane potential (RMP) (Control: n = 16 neurons from 3-4 mice; *Foxg1* cKO: n = 16 neurons from 3-4 mice) and input resistance (Control: n = 13 neurons from 3-4 mice; *Foxg1* cKO: n = 12 neurons from 3-4 mice) measurements, showing no genotype-specific differences. **D** Action potential (AP) frequency across stimulus intensities, confirming comparable intrinsic excitability (Control: n = 15 neurons from 3-4 mice; *Foxg1* cKO: n = 15 neurons

from 3-4 mice). **E** Neuronal AP threshold was unaffected by FOXG1 loss in iSPNs (Control: *n* = 12 neurons from 3-4 mice; *Foxg1* cKO: *n* = 13 neurons from 3–4 mice). Data are presented as the mean ± SEM. Statistical analysis by unpaired two-tailed *t*-test for all comparisons, except for panel **D**, which employed the two-way repeated measures ANOVA followed by Slidák’s multiple comparisons test; ns, not significant; cKO, conditional knockout.

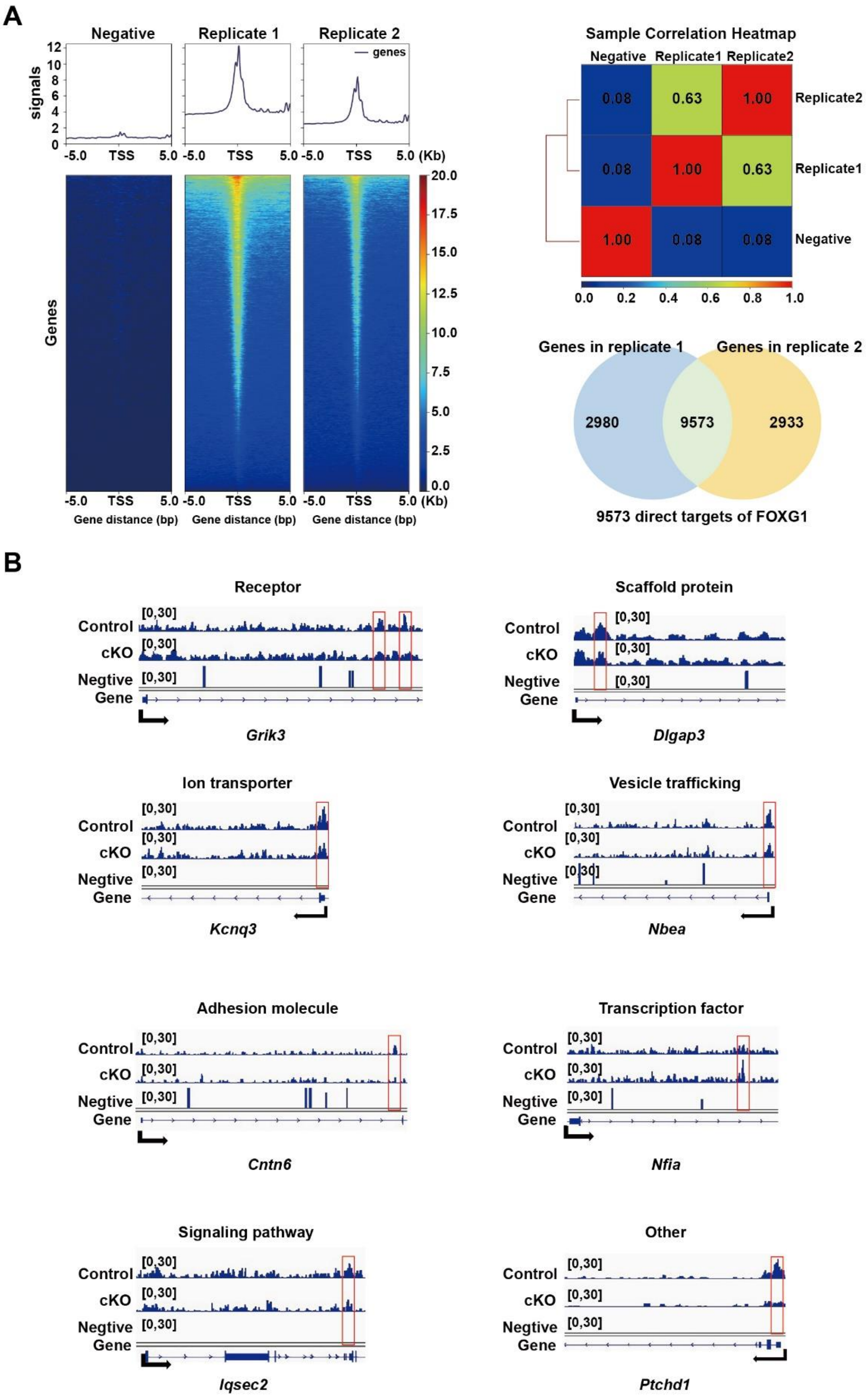
A
Negative
Replicate 1
Replicate 2
genes
signals
TSS
Genes
Gene distance (bp)
Sample Correlation Heatmap
Negative Replicate1 Replicate2
0.08 0.63 1.00 Replicate2
0.08 1.00 0.63 Replicate1
1.00 0.08 0.08 Negative
Genes in replicate 1
Genes in replicate 2
2980
9573
2933
9573 direct targets of FOXG1
B
Receptor
Grik3
Scaffold protein
Dlgap3
Ion transporter
Kcnq3
Vesicle trafficking
Nbea
Adhesion molecule
Cntn6
Transcription factor
Nfia
Signaling pathway
Iqsec2
Other
Ptchd1
Control
cKO
Negtive
Gene
[0,30]

Fig. S2 Identification of potential FOXG1 binding sites at synapse-related gene loci by IGV peak plot. **A** Analyses of CUT&Tag-seq in control mice striatal cells at P30. Heatmaps showing the CUT&Tag signals 5.0 kb upstream and 5.0 kb downstream of the transcription start sites in control replicates 1&2 (FOXG1 antibody was used), and negative control (no antibody). Hierarchically clustered correlation matrix of the negative control, replicate 1&2. Venn plot: the number of shared genes and unique genes of replicates 1&2. **B** Integrative Genomics Viewer (IGV) peak plots identify the examples of putative FOXG1 binding sites in functionally diverse synaptic targets, including: receptors (*Grik3*), scaffolding protein genes (*Dlgap3*), ion channels (*Kcnq3*), vesicle trafficking gene (*Nbea*), adhesion molecules (*Cntn6*), transcription factor *Nfia*, signaling molecule *Iqsec2*, and other *Ptchd1*. Regions highlighted in red indicate putative FOXG1 binding loci. cKO, conditional knockout.

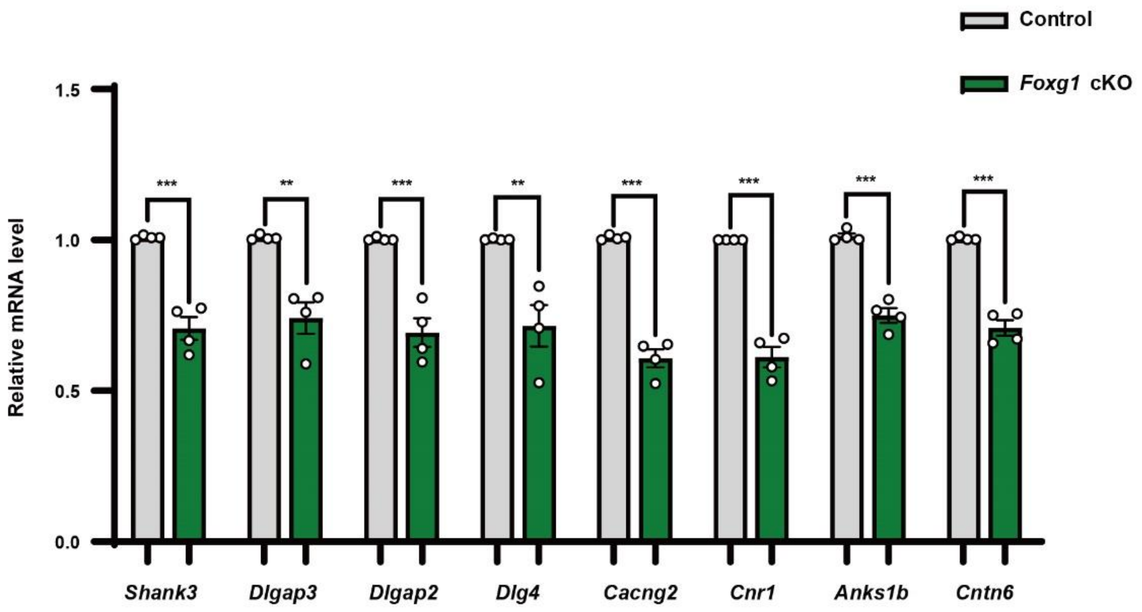

Fig. S3 *Foxg1* deletion in iSPNs induces abnormal mRNA expression of synaptic-related genes. Quantitative PCR analyses reveal significantly reduced transcriptional expression of the synaptic-related genes in *Foxg1* cKO iSPNs compared to control iSPNs (n = 4 biological replicates). Data are

presented as the mean ± SEM. Statistical analysis by unpaired two-tailed *t*-test for all comparisons. $^{**}P<0.01$, $^{***}P<0.001$. cKO, conditional knockout.

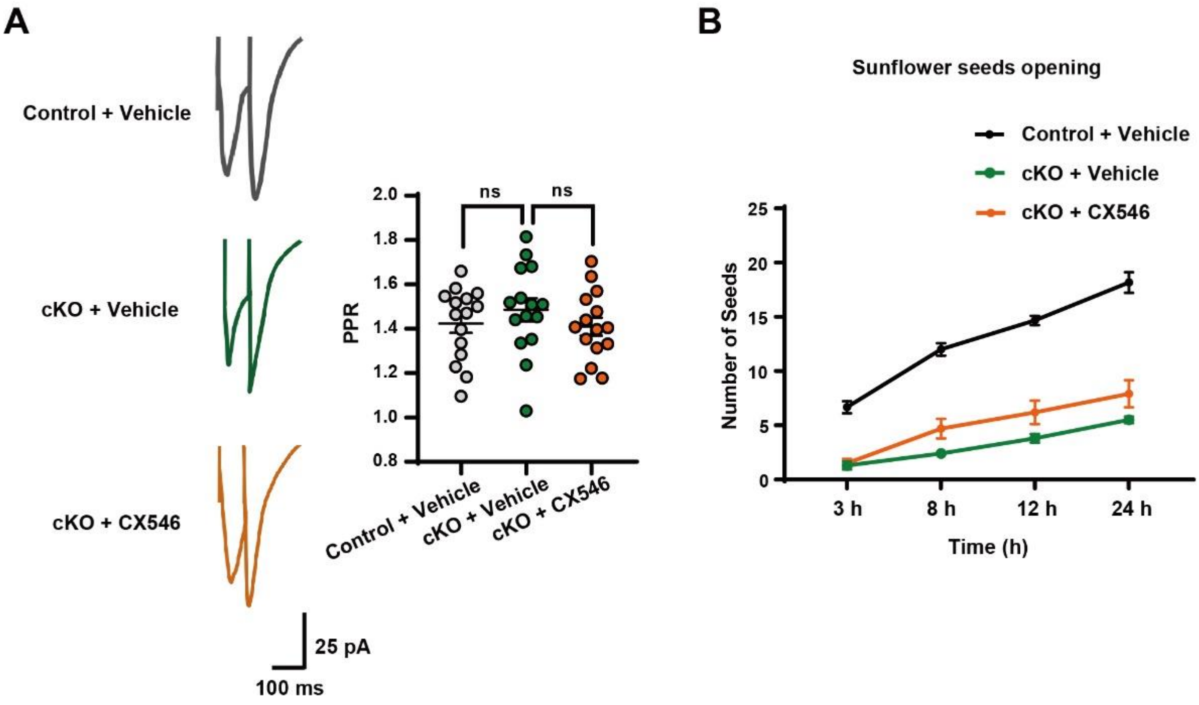

Fig. S4 CX546 fails to rescue the PPR and sunflower seeds opening task. **A** Paired-pulse ratio (PPR) remains unchanged in iSPNs following CX546 treatment (n = 15 neurons from 3–4 mice per group). **B** The sunflower seed-opening task shows a non-significant trend toward improvement in CX546-treated *Foxg1* cKO mice (Control + Vehicle: $n = 6$; *Foxg1* cKO + Vehicle: $n = 10$; *Foxg1* cKO + CX546: $n = 10$). Data are presented as the mean ± SEM. One-way ANOVA followed by Tukey's multiple comparisons test for panel **A**, Friedman's M-test for panel **B**. ns, not significant; cKO, conditional knockout.

Supplemental Tables

Table S1 The primers for *in situ* probes and qPCR

Gene name	Sequence (5'→3')
Mouse *Drd2* (*in situ*)	F: agctccaccgcggtggcggccgcCGGGAGCTGGAAGCCTCG
	R: cttgatatcgaattcctgcagTGCAGGGTCAAGAGAAGGCC
Mouse *Adora2a* (*in situ*)	F: AAATATGCGGCCGC ATGGCTCCTCGGTGTACATCATG
	R: ACGCGTCGAC TCAGGAAGGGGCAAACTCTGAAGAC
Mouse *Penk* (*in situ*)	F: tcccccgggctgcaggaattcTCGGAAGGACAGGATGTCATCA
	R: gggccccccctcgaggtcgacCGTCAGGAGAGATGAGGTAACAAA

Table S2 The primers for qPCR

Gene name	Sequence (5'→3')
Mouse *Adora2a* (qPCR)	F: GATGGAGAGCCAACCCCTAC
	R: TTGATGATGTGCAGGGGCAA
Mouse *Anks1b* (qPCR)	F: CTCAGGGAACCCAGTGGTAAT
	R: GCATGATAATGTGAGGAACGTCT
Mouse *Cacng2* (qPCR)	F: CAAGTTCCCATTCAATACCCAGT
	R: AACATGGTGCATCCATACATAGG
Mouse *Cnr1* (qPCR)	F: TCTGCTTGCGATCATGGTGT
	R: GCATGTCTCAGGTCCTTGCT
Mouse *Cntn6* (qPCR)	F: GCAGGTGAGGGTCGTTTCAG
	R: GGAGAAGGGTATCCATTTGCAG
Mouse *Dlg4* (qPCR)	F: TATCGCCATCTTCATCCGTCC
	R: CTCTTCAAAGCTGTCGCCCT
Mouse *Dlgap2* (qPCR)	F: GCGGCAGCCTTATCTCCTTAG
	R: GGATACGTGGTCACCCAACAT
Mouse *Dlgap3* (qPCR)	F: GAGGGTCCATCATCAGTAGGC
	R: CGCTGGTATGGGAGTGTGTG
Mouse *Drd2* (qPCR)	F: GTTTCTGTACCCTTCGGGGG
	R: AGAAGTGGCGGAGTGGATTG
Mouse *Gria1* (qPCR)	F: GGACAACTCAAGCGTCCAGA
	R: GTCGGTAGGAATAGCCCACG
Mouse *Gria2* (qPCR)	F: TTCTCCTGTTTTATGGGGACTGA
	R: CCCTACCCGAAATGCACTGTA
Mouse *Gria3* (qPCR)	F: GGGGTTTGGTGGTTCTTCAC
	R: ACAGATGGCTCTGCGGATTTC
Mouse *Penk* (qPCR)	F: AAGAGCTACTGGGAACGGGA

	R: ATCTTCCAGTTGGGGGCTTC
Mouse *Shank3* (qPCR)	F: CATTGGGGAGGGCGGTTTCT
	R: CTCTGGTTTCATGCCGGGT
Mouse *GAPDH* (qPCR)	F: AACTTTGGCATTGTGGAAGGG
	R: GACACATTGGGGGTAGGAACA

Table S3 The primers for vector construction of luciferase assay

Gene name	Sequence (5'→3')
Mouse *Gria1*	F: cgagctcttacgcgtgctagcTTAAAAATATAGCAACAATATTTAAAAGTTCA
	R: cagtaccggaatgccaagcttTAGATTTGGTCTTCCCTCCCCT
Mouse *Gria2*	F: cgagctcttacgcgtgctagcACAAAATATCCCCTCGTTTCCC
	R: cagtaccggaatgccaagcttATGTAACAAGTTGGGGTATTTATAATTCT
Mouse *Gria3*	F: cgagctcttacgcgtgctagcTAATGAATTAGATGCTTTTACACCC
	R: cagtaccggaatgccaagcttACCAATCTGGAAGGCGGG
